# Medical trainees' emotions and their effects on perceptions of performance and team mood in team‐based simulations

**DOI:** 10.1111/bjep.70017

**Published:** 2025-08-13

**Authors:** Keerat Grewal, Sayed Azher, Matthew Moreno, Reinhard Pekrun, Jeffrey Wiseman, Jessica Flake, Susanne Lajoie, Ning‐Zi Sun, Gerald M. Fried, Elene Khalil, Jason M. Harley

**Affiliations:** ^1^ Department of Surgery McGill University Montreal Quebec Canada; ^2^ Department of Psychology University of Essex Colchester UK; ^3^ Department of Psychology & Pedagogy Ludwig Maximilian University of Munich Munich Germany; ^4^ Institute for Positive Psychology and Education Australian Catholic University Sydney New South Wales Australia; ^5^ Institute of Health Sciences Education McGill University Montreal Quebec Canada; ^6^ Steinberg Centre for Simulation and Interactive Learning (SCSIL) McGill University Montreal Quebec Canada; ^7^ Department of Psychology McGill University Montreal Quebec Canada; ^8^ Department of Educational and Counselling Psychology McGill University Montreal Quebec Canada; ^9^ McGill University Health Centre for Interprofessional Simulation Montreal Quebec Canada; ^10^ Research Institute of the McGill University Health Centre Montreal Quebec Canada; ^11^ Present address: Department of Psychology The University of British Columbia Vancouver British Columbia Canada

**Keywords:** emotions, medical education, mixed‐methods, simulation

## Abstract

**Background:**

Emotions affect performance in learning contexts; however, their effects on medical trainees' performance in highly ecologically valid settings, like team‐based simulation training, are not well understood. It is therefore imperative to know which emotions are experienced by medical trainees and the impacts of these emotions on perceptions of performance and team mood.

**Aims:**

To extend the understanding of medical trainees' emotions in the context of team‐based medical simulations using a new self‐report tool (Situated Emotion Regulation Questionnaire; SERQ).

**Sample:**

Participants were 106 medical trainees participating in team‐based simulations. Seventy‐one participated in multiple simulations.

**Methods:**

A field‐based, mixed‐methods methodology was used. Medical trainees self‐reported their emotions and perceptions of individual performance, team performance and team mood. Multi‐level analyses were used to account for nestedness. Debriefings were qualitatively analysed to provide validity evidence for the SERQ.

**Results:**

Team leaders reported significantly higher levels of shame post‐simulation than team members. A variable comprising post‐simulation happiness and hopefulness was a significant predictor of perceptions of team performance and team mood. Post‐simulation frustration was a significant predictor of perceptions of team mood. Participants' SERQ responses demonstrated alignment or mixed alignment with their debriefing responses.

**Conclusion:**

Using multi‐level analyses, our research provides insight into medical trainees' emotions and their effects on perceptions in highly ecologically valid simulation trainings. Future medical education training may use these findings to develop curricula and simulations to induce specific emotions or practice emotion regulation. Additionally, the SERQ demonstrated promising validity evidence and may be a valuable future research and educational tool.

## INTRODUCTION

Emotions can affect psychophysiological processes, thereby influencing learning and performance (Camacho‐Morles et al., 2021; Frenzel et al., 2023; Gross, 2024). Higher education learners reporting pleasant emotions (e.g. happiness) are typically more engaged, perform better and attend class more than students reporting unpleasant emotions (e.g. frustration) (Earl et al., 2024; Parker et al., 2021; Pekrun et al., 2023; Robinson et al., 2017; Tze et al., 2022; Wortha et al., 2019). However, emotional effects are nuanced. For example, Jarrell et al. (2016) found that students experiencing pleasant emotions rated their performance higher, despite no objective performance differences from those experiencing unpleasant emotions. These findings suggest that emotional valence can influence self‐perceptions differently from objective outcomes, highlighting the need to further investigate the role of emotions within specific contexts (Camacho‐Morles et al., 2021; Gross, 2024; Pekrun & Linnenbrink‐Garcia, 2022).

For example, medical trainees in simulation‐based educational environments may have their emotions influenced by many factors, including if they are the team leader (in the ‘hot seat’; their performance is usually evaluated) or team member and the information they are given beforehand (Duque et al., 2023; Harley & Pekrun, 2024; Mueller et al., 2022). Since well‐designed simulations replicate realistic environments to prepare medical trainees for emotion‐evoking situations, trainees may experience simulations and related emotions as if facing real cases (Gaba, 2004; Macdougall et al., 2013). Emotions can also spread to other team members through mechanisms such as emotional contagion, where one person's emotional state can influence the emotions of others, affecting team performance and mood (Herrando & Constantinides, 2021; Mitchell & Boyle, 2019; Yang et al., 2021). Compared to emotions, moods typically last longer, are less intense and ‘often do not have specific object [foci]’ (Gross, 2024). Positive team moods can enhance teamwork, thereby improving quality of care and patient safety (Forsyth, 2021; Rosen et al., 2018; Varpio & Teunissen, 2021).

Post‐simulation debriefings allow trainees to reflect on their performance and experiences in the simulation and/or discuss the scenario's real‐life applicability (Sawyer et al., 2016). Many debriefing frameworks dedicate a phase to discuss trainees' emotions and reactions (e.g. Bajaj et al., 2018; Kolbe et al., 2013; Zigmont et al., 2011). Understanding trainees' simulation‐induced emotions is essential, as these may influence decision‐making and their team's emotions (Herrando & Constantinides, 2021; LeBlanc & Posner, 2022; Madsgaard et al., 2022; Yang et al., 2021).

This study uses a novel self‐report tool to examine emotions in team‐based medical simulations. Emotions immediately before and after each simulation were the foci of the study as collecting self‐report data on emotions during the simulation would interfere with the educational opportunity. As such, this study examines if participants' roles during simulations influence the emotions experienced after the simulation while controlling for emotions before the simulation, the relationship of post‐simulation emotions with performance and team mood, and provides validity evidence for the self‐report tool. This study also explores how participants discuss their emotions and perceptions during debriefings, which has not previously been done in the medical simulation training context. The theoretical frameworks that guided our study and a brief overview of the literature of emotions in medical education are presented below, followed by a brief overview of the current study.

### Theoretical framework

We used the control‐value theory of achievement emotions (CVT; Pekrun, 2024), which is widely used in academic achievement research (Shao et al., 2023; Tze et al., 2023). CVT characterizes emotions by pleasantness (*valence*; positive to negative), degree of physiological arousal (*activation*; activating to deactivating), the stimuli triggering the emotion (*object focus*; task‐related or outcome‐related) and the place in time the emotion was directed to (*time frame*; retrospective, concurrent or prospective; Pekrun et al., 2023). For example, a cardiac arrest simulation may induce frustration triggered by missing equipment (concurrent, activity‐related).

Positive activating emotions (e.g. excitement) are typically associated with increased achievement, whereas negative deactivating emotions (e.g. hopelessness) are associated with impaired achievement (Madsgaard et al., 2022; Pekrun & Linnenbrink‐Garcia, 2022; Vogl et al., 2020). However, some studies have found evidence that diverges from this general pattern (e.g. Jarrell et al., 2016; McConnell et al., 2016). For example, McConnell and colleagues found that students experiencing either positive or negative emotions when learning about physiological concepts performed worse than those who were in an emotionally more neutral state, suggesting that heightened emotional intensity, regardless of valence, may interfere with cognitive processing. Jarrell and colleagues found that students who experienced positive emotions had similar objective performance on a diagnostic reasoning task compared to those experiencing negative emotions. As predicted by CVT, these findings challenge the assumption that positive emotions universally enhance and negative emotions impair performance (Pekrun, 2006, 2024). The findings indicate that there is a need to explore the effects of different emotions within the categories of positive and negative emotions. They also suggested that research should attend to possible differences of emotion effects across different academic tasks, different learner populations and different contexts, as the findings of previous studies may not be generalizable to all tasks, populations and contexts. As such, identifying the differences in emotion effects for medical learners engaging in team‐based medical simulations extends and addresses a gap within the literature.

We used *The Standards for Educational and Psychological Testing* (American Educational Research Association, American Psychological Association, & National Council on Measurement in Education, 2014) to guide validity evidence collection and argumentation for the self‐report tool, particularly for evidence based on *content*, *internal structure*, *response processes* and *relations to other variables* (descriptions can be found in Data S1: Material I).

### Trainees' emotions in medical simulations

Literature regarding emotions in medical simulations is often limited to only negative activating emotions (e.g. anxiety), which may be due, in part, to medical simulations being designed to allow trainees to practise managing specific negative emotions that are commonly experienced in real‐life scenarios (Ahn et al., 2023). However, simulations can also evoke positive and deactivating emotions (Keskitalo & Ruokamo, 2021; Madsgaard et al., 2022; Yang et al., 2021). For example, Keskitalo and Ruokamo (2021) found that medical trainees experienced higher levels of positive emotions and lower levels of negative emotions post‐simulation compared with presimulation.

Additionally, previous studies do not account for the nesting (e.g. trainees nested in teams) often present during simulation training, which may lead to errors in interpreting results (Leppink, 2015; Zyphur et al., 2008), especially for studies examining the effects of emotions on performance and learning (e.g. Behrens et al., 2019; Duffy et al., 2015, 2020). Thus, the understanding of the role(s) of emotions in team‐based medical simulations is incomplete as the nesting effects of the team and simulation scenario have not been considered. Trainees may respond differently across teams and simulation scenarios. Before understanding how emotions affect performance and team dynamics in simulation training, we need to identify the emotions experienced in team‐based simulations while accounting for the effects of nesting caused by team compositions and simulation scenarios.

### Research objectives

We examined medical trainees' emotions and their effects on perceived performance and team mood during nested, team‐based medical simulations. To do so, we developed and collected the first evidence of validity for a novel self‐report tool (Situated Emotion Regulation Questionnaire; SERQ) capable of examining current emotional states, and perceptions of performance and team mood in one self‐report measure. We sought to answer the following research questions (RQs):

RQ1. Which current emotions are reported by medical trainees after participating in a team‐based medical simulation?
*Expectation*: As simulations are designed to be realistic, it is anticipated that medical trainees report experiencing a wide range of emotions after a simulation (positive, negative, activating and deactivating emotions; Madsgaard et al., 2022).
RQ2. When accounting for nesting caused by variations in simulation scenarios, team compositions and repeated observations from individuals, how does medical trainees' reported intensity of current emotions after a team‐based medical simulation vary between team leaders and team members?
*Hypothesis*: As team leaders are usually evaluated by medical experts as part of their curriculum, it is expected that team leaders will have higher intensities of negative activating emotions (Madsgaard et al., 2022).
RQ3. When accounting for nesting (simulation, team, individual), do the current emotions after a team‐based medical simulation predict medical trainees' perceived (a) team mood, (b) team performance or (c) individual performance?
*Hypothesis*: Positive emotions are expected to increase trainees' perceptions of performance and team mood as trainees may be more overconfident in their perceived simulation performance and team dynamics due to their current emotional state (Kensinger & Ford, 2020). Negative emotions are expected to impair perceptions as trainees may recall their errors during the simulation more accurately (Kensinger & Ford, 2020). Deactivating emotions are expected to be experienced at low intensities due to the high‐stakes nature of the simulations and are therefore expected to have minimal effects on participants' perceptions (Macdougall et al., 2013; Pekrun, 2024).
RQ4. How do medical trainees discuss (a) emotions, (b) perceived performance and (c) team mood during post‐simulation debriefings?
*Hypothesis*: As debriefings provide participants with a chance to reflect on their performance and experiences in the simulation, including their emotional reactions, we anticipated that participants would also discuss their concurrent emotions and perceptions of performance and team mood following the simulation.
RQ5. How aligned are medical trainees' reported emotions and perceptions of performance between their self‐report tool responses and their post‐simulation debriefing responses?
*Hypothesis*: Emotions reported in the SERQ are expected to capture medical trainees' emotions and therefore be in alignment with their responses during the debriefing period regarding their emotions. Perceptions of performance reported in the SERQ are expected to be in alignment with medical trainees' debriefing responses regarding their performance.

## METHODS

A field‐based, observational, mixed‐methods study was conducted in a medical simulation training environment. Such a study is akin to a classroom study, but for physicians in training. The SERQ was completed immediately before and after the team‐based medical simulation. Audio‐video data were collected from simulations and debriefings. Informed consent was collected for all participants.

### Participants

Data were collected from 106 residents (medical trainees) at two simulation centres at a North American medical university and affiliated hospital (Appendix A). Data were collected from 69 teams and 69 simulations. There was variance in how many teams participated in each simulation. Teams were not assigned by the researchers to minimize any interference with the simulation educational opportunity. Teams comprised of 2–8 residents (*M* = 3.37), typically with one leader. 71 residents participated in multiple simulations, playing the same or different roles, resulting in multiple datapoints for these participants. Overall, there were 242 datapoints collected from participants. From these, 79 datapoints were from leaders, while 163 were from team members. After applying listwise deletion to remove cases with missing data (*n*_datapoint_ = 69; 28.5%), 173 datapoints from 85 participants were used for analyses (see Data S1: Material II).

### Simulation environment

Presimulation, medical trainees were assigned or elected to be leaders or team members. Leaders were summatively assessed by medical experts, though these data were not collected. Simulations used high‐fidelity manikins (capable of responding to actions) with some also using confederates (e.g. medical professionals role‐playing in the simulation). On average, simulations were 14 minutes and 20 s long, ranging from 5 to 36 min. Debriefings were held after each simulation by medical experts (debriefers) where simulation content was discussed (*M* = 27 min and 21 s, range: 6–59 min). Audio‐video equipment present in the simulation centres recorded the simulation scenarios and debriefings.

The study did not interfere with the manner in which simulations were used in the curriculum. Thus, there was heterogeneity in the simulations, including the team size and difficulty, as well as the debriefings, including the number of debriefers, the quality of the debriefings and the debriefing framework (if any were used at all).

### Situated Emotion Regulation Questionnaire (SERQ)

For information regarding the development goals of the SERQ, see Data S1: Material III. The SERQ provides a narrative‐style summary of participants' responses (Appendix B) that is automatically emailed to participants to encourage reflection and potentially increase the quality of the emotions phase of debriefings (beyond the scope of this study, but a future aim). The SERQ (Data S1: Material IV) includes a modified version of the Medical Emotion Scale (MES; Duffy et al., 2020), drawing upon 9 items and adding 2 more items (stress and nervousness). Further justification and validity evidence the SERQ draws from can be found in Data S1: Material I.

Performance‐ and team mood‐related items were adapted from Webster and Hadwin's Socio‐Emotional Sampling Tool (2014) and Socio‐Emotional Reflection Tool (2012) to fit a medical education context. This allowed participants to receive a self‐narrative‐style summary immediately after completing the SERQ that would capture their perceptions and possibly support further self‐reflection. The adapted items were single‐item responses which allowed for brevity to accommodate the logistical constraints of medical training. Four external medical experts provided feedback on the SERQ during its development.

In the SERQ, participants indicated the intensity (i.e. ‘Please indicate how you feel right now […]’ for each affective state; 1 = not at all, 2 = a little, 3 = moderately, 4 = strongly, 5 = very strongly) of 11 affective states while thinking about the simulation that was upcoming (pre‐SERQ) or had already been completed (i.e. after the simulation; post‐SERQ). The affective states were: curiosity, confusion, shame, relief, stress, frustration, hopelessness, happiness, hopefulness, pride and nervousness. Due to limited sample size and power constraints, item removal and reduction were conducted, reducing the number of items to 7 emotions (see Data S1: Material V–IX for justification). The items used for the analyses were: curiosity, confusion, shame, frustration, pride, nervousness and a variable comprising the average of ratings of happiness and hopefulness.

The *post*‐SERQ also measures participants' perceptions of *team performance* (i.e. satisfaction with the team's performance; 1 = very unsatisfied, 5 = very satisfied), *individual performance* (i.e. satisfaction with their own performance; 1 = very unsatisfied, 5 = very satisfied) and *team mood* during the simulation (1 = very negative, 5 = very positive). It also contains items related to emotion regulation strategies; however, these were not used in the presented analyses because they were beyond the scope of our research questions.

### Qualitative content analysis: Source of validity evidence (RQs 4–5)

We expected participants' spontaneous debriefing responses related to their current emotions post‐simulation and perceptions of individual performance, team performance and team mood to align with their post‐SERQ responses. Qualitative content analysis was conducted in accordance with Mayring's (2014) guidelines to determine if participants' responses were aligned between the debriefings and self‐reports to ascertain the first evidence of validity for the SERQ. From 69 debriefings, 13 were identified where all participants had consented and provided audio‐video and self‐report data. Notably, 13 of the 32 participants were in multiple debriefings. The first author deidentified the debriefing audios. Trained undergraduate volunteers transcribed the deidentified audios, and the first author reviewed the transcriptions. The first author, in consultation with the senior author, iteratively developed and applied the coding scheme to the transcriptions.

Codes were developed deductively and inductively, using items in the SERQ and any debriefing responses that were not in the SERQ (Appendix C). Details related to observer/debriefer coding and findings can be found in Data S1: Material X.

An observation was defined as the presence of a specific code for each participant during a debriefing. For example, if a participant had 2 ‘confused’ codes during one debriefing and 3 ‘confused’ codes for another debriefing, they would be considered 2 ‘confused’ observations as they had ‘confused’ codes present across two different debriefings. Observations were compared with SERQ responses. In total, 40 observations from 32 participants were used for analysis.

Comments about team mood or performance made by a team member regarding another member of the team were considered proxies for perceptions of team mood or team performance, even if the comment was made only in reference to a single member rather than the entire team (Shapiro et al., 2008). Performance‐ and team mood‐related observations were based on the number of positively‐ and negatively‐oriented codes for each participant. Participants were categorized into ‘positive perceptions’, ‘negative perceptions’ and ‘mixed perceptions’ observations based on verbal statements made across the entire debriefing session. Participants categorized into ‘positive perceptions’ had greater than 50% of their coded debriefing statements be positively‐oriented. For example, if a participant had expressed 3 statements during the debriefing indicating that they believed they performed well (positively‐oriented response) and one statement indicating that they believed they did not perform well (negatively‐oriented response), they would be categorized as having ‘positive perceptions’ since 75% of their debriefing statements were positively‐oriented. ‘Negative perceptions’ were those with greater than 50% of their debriefing statements coded as negatively‐oriented. ‘Mixed perceptions’ had mixed debriefing responses and included those with 50% or less of their statements coded as negatively‐ or positively‐oriented. For example, if a participant had 2 statements during the debriefing indicating that they believed they performed well, 2 statements indicating they believed they performed poorly and 1 statement where it was unclear how they felt about their performance (e.g. a neutral statement), they were categorized as ‘mixed perceptions’ as < 50% of their statements were positively‐ or negatively‐oriented.

Information on alignment can be found in Data S1: Material XI.

### Statistical analysis (RQs 1–5)

**FIGURE 1:**
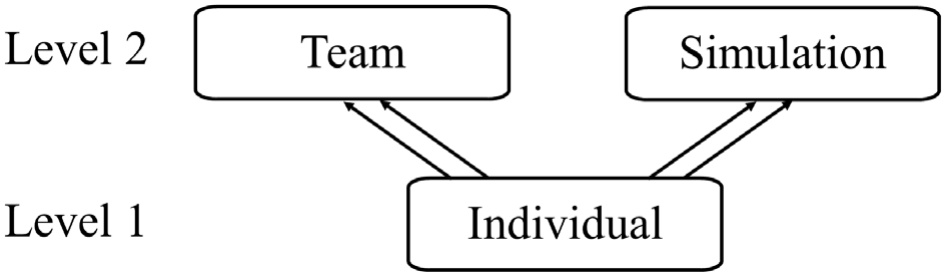
Schematic of the multiple membership model of the dataset. Arrows indicate the nesting present in the dataset. Double arrows indicate multiple membership. Level 1 (individuals) is nested within level 2 (teams and simulations).

Data were analysed using Stata BE 18.0 (StataCorp, 2023) and IBM SPSS Statistics, Version 28. All Likert‐type scale responses (1–5 or 1–7) were treated as continuous, as literature suggests that Likert‐type scales with five or more categories can reasonably approximate interval‐level data (Norman, 2010). To account for simulation and team heterogeneity and ensure type I error rates were not inflated from downwardly biased standard errors, multi‐level modelling analyses with a cross‐classified, multiple membership design were used, with simulation (e.g. unique simulation scenarios) and team (e.g. the unique composition of individuals within teams) as the second levels, and individuals (e.g. individual participants) as the first level (Figure 1). When the variance accounted for by the nesting was negligible for certain levels (ICCs < .05), those levels were removed from the final models. No multi‐collinearity was found between the independent variables across all models (Data S1: Material XII–XIII). Multi‐level models with additional covariates (gender and racialized minority status) were also conducted (Data S1: Material XIV) but raised concerns about power, as there were an inadequate number of observations for the number of variables in the model. Bonferroni corrections were used to account for family‐wise error rates. Codes for analyses are provided in Data S1: Material XV.

## RESULTS

### Medical trainees' reported emotions (RQ1)

Descriptive statistics are provided in Table 1. Compared to team members after a team‐based medical simulation, leaders reported descriptively higher mean intensities of happiness, pride, relief and all negatively valenced emotions than team members. Conversely, the mean intensities for curiosity and hopefulness were lower for leaders than for team members.

**TABLE 1:** Descriptive statistics of self‐reported emotion intensities.

	Leader (*N* = 24; obs = 57)		Member (*N* = 61; obs = 116)	
	*M*	SD	*M*	SD
**Presimulation**				
Positive activating emotions				
Curiosity	3.35	.97	3.19	.98
Happiness	2.60	.90	2.78	.96
Hopefulness	2.72	.96	2.83	.99
Pride	2.32	.98	2.52	1.16
Positive deactivating emotions				
Relief	1.75	.83	2.03	1.06
Negative activating emotions				
Confusion	2.04	.87	1.95	.89
Shame	1.60	.82	1.50	.84
Stress (affective state)	3.05	.95	2.49	.95
Frustration	1.58	.78	1.50	.73
Nervousness	3.19	1.13	2.62	1.04
Negative deactivating emotions				
Hopelessness	1.49	.76	1.48	.76
**Post‐simulation**				
Positive activating emotions				
Curiosity	2.79	1.06	2.97	1.08
Happiness	2.88	.85	2.78	1.01
Hopefulness	2.77	.96	2.90	1.07
Pride	2.70	.82	2.64	1.05
Positive deactivating emotions				
Relief	3.23	1.07	2.84	1.06
Negative activating emotions				
Confusion	2.39	.98	2.21	1.05
Shame	2.09	1.01	1.60	.91
Stress (affective state)	2.47	.98	2.05	.88
Frustration	2.18	1.05	1.85	1.02
Nervousness	2.35	.79	2.10	.97
Negative deactivating emotions				
Hopelessness	1.63	.86	1.47	.79
**Individual performance**	3.12	.71	3.19	.83
**Team performance**	3.58	.78	3.53	.87
**Team mood**	3.77	.71	3.81	.78

*Note*: Means and standard deviations are based on total datapoints (observations; obs). Data are from 69 teams and from 69 different simulations.

### Comparing leaders' and team members' emotions (RQ2)

Seven multi‐level model analyses were conducted to determine differences in emotions between team leaders and team members after a team‐based medical simulation. The multi‐level models were constructed with the emotion as the dependent variable at the individual level (null and predictor‐only models: Appendices D and E). For all full models, simulation role (i.e. team leader vs. team member) was entered as a predictor while presimulation emotion intensity, training level (i.e. PGY) and specialty (e.g. internal medicine, anaesthesiology) were entered as covariates (Table 2). To account for the family‐wise error rate using Bonferroni corrections, significance was defined at the *p* < .007 level. The post‐simulation shame model indicated significant differences between team leaders and team members. Specifically, team leaders reported higher levels of shame after a simulation as compared to team members (*b* = .38, *p* = .001, *η* ^2^ = .04, power = .99).

**TABLE 2:** Multi‐level models of the effect of simulation role on post‐simulation emotions with covariates.

	Curiosity	Confused	Ashamed	Frustrated	Proud	Nervous	Happy and hopeful
Intercept	1.462**	2.426**	1.297**	1.289**	1.331**	1.638**	−.077
Simulation role	−.226	.049	.383*	.285	.154	.008	.155
Presimulation emotion	.415**	.191	.503**	.529**	.534**	.306**	.604**
PGY	.067	−.114	−.068	−.027	.018	−.114	−.111
Specialty							
Internal medicine	.112	−.178	−.348	.023	−.142	.223	.153
Emergency medicine	.088	−.367	−.157	−.381	−.040	−.303	.554
Critical care	−.388	−.273	−.448	−.356	−.189	.423	.837
OBGYN	−.039	−.070	−.061	−.182	−.169	.240	.427
Variance components							
Simulation variance	–	.263	.195	–	–	.072	–
Team variance	–	–	–	.048	–	–	–
Individual variance	.097	.066	.040	.124	<.001	.183	.003
Pseudo *R* ^2^	.096	.063	.272	.207	.340	.332	.385

*Note*: The full models were significant, χ^2^(7) ≥ 29.63, *p* < .05. Simulation role refers to participants role in the simulation as team member (assigned a value of 0) or team leader (assigned a value of 1). Presimulation emotion refers to the intensity of the emotion of interest measured before the simulation. PGY refers to the post‐graduate year of the participant (i.e. training level). All specialty coefficients compare the specialty of interest (assigned a value of 1) to participants specializing in anaesthesia (assigned a value of 0). Anaesthesia was selected as it had the highest number of datapoints from all other specializations. OBGYN refers to the obstetrics and gynaecology specialty. The variance components represent the random effects of the model. Simulation variance refers to the variance estimate at the simulation level. Team variance refers to the variance estimate at the team level. Individual variance refers to the variance estimate at the individual participant level. AIC and BIC values can be found in Data S1: Material XVI. *p < .007, **p < .001.

### Individual performance predictors (RQ3)

A multi‐level model analysis was conducted to determine whether emotion intensities after the simulation are predictors of perceived individual performance for medical trainees. Post‐simulation curiosity, confusion, shame, frustration, pride, nervousness and a happiness and hopefulness variable (see Data S1: Material V–IX for more information on these variables) were entered as predictors while presimulation emotions, simulation role, training level and specialty were entered as covariates. To account for the family‐wise error rate, significance was defined at the *p <* .007 level. The results of the null, predictor‐only and full model are reported (Table 3). While the predictor‐only model revealed that post‐simulation pride was a significant positive predictor of perceived individual performance (*b =* .25, *p* = .001, *η* ^2^ = .06, power > .99); once covariates were added to create the full model, and no post‐simulation emotion was a significant predictor of perceived individual performance.

**TABLE 3:** Multi‐level models of the effect of post‐simulation emotions on perceptions of individual performance.

	Null	Null + post‐simulation emotions	Full model with covariates
Intercept	3.163**	3.215**	3.081**
Post‐simulation emotions			
Curiosity		.003	−.005
Confused		−.069	−.061
Ashamed		−.041	−.101
Frustrated		−.178	−.160
Proud		.251*	.187
Nervous		−.060	−.012
Happy + Hopeful		.020	.094
Presimulation emotions			
Curiosity			−.007
Confused			.064
Ashamed			.047
Frustrated			−.091
Proud			.168
Nervous			−.052
Happy+Hopeful			−.170*
Simulation role			.041
PGY			.005
Specialty			
Internal medicine			−.121
Emergency medicine			.080
Critical care			−.372
OBGYN			−.120
Variance components			
Team variance	.073	.021	<.001
Individual variance	.046	.016	<.001
ICC_T_	.114		
ICC_I>T_	.186		

*Note*: The full model was significant, χ^2^(20) = 109.77, *p* < .001, pseudo *R* ^2^ = .392. Simulation role refers to participants role in the simulation as team member (assigned a value of 0) or team leader (assigned a value of 1). PGY refers to the post‐graduate year of the participant (i.e. training level). All specialty coefficients compare the specialty of interest (assigned a value of 1) to participants specializing in anaesthesia (assigned a value of 0). Anaesthesia was selected as it had the highest number of datapoints from all other specializations. OBGYN refers to the obstetrics and gynaecology specialty. The variance components represent the random effects of the model. Team variance refers to the variance estimate at the team level. ICC_T_ refers to the intraclass coefficient for the team level. Individual variance refers to the variance estimate at the individual participant level. ICC_I>T_ refers to the intraclass coefficient for the individual level nested within the team level. AIC and BIC values can be found in Data S1: Material XVII. *p < .007, **p < .001.

### Team perception models (RQ3)

The following analyses used multi‐level model analyses. Post‐simulation curiosity, confusion, shame, frustration, pride, nervousness and a happiness and hopefulness variable were entered as predictors while presimulation emotions, simulation role, training level and specialty were entered as covariates. To account for the family‐wise error rate, significance was defined at the *p* < .007 level.

#### Team performance predictors

A multi‐level model analysis was conducted to determine whether emotion intensities after the simulation are predictors of perceived team performance for medical trainees. The results of the null, predictor‐only and full model are reported (Table 4). From the model, the post‐simulation happiness and hopefulness variable was a significant predictor at the individual level for perceptions of team performance. As scores for the post‐simulation happiness and hopefulness variable increased, perceptions of team performance increased, *b =* .22; *p* = .001; *η* ^2^ = .08; power > .99.

**TABLE 4:** Multi‐level models of the effect of post‐simulation emotions on perceptions of team mood.

	Null	Null + post‐simulation emotions	Full model with covariates
Intercept	3.779**	4.631**	4.810**
Post‐simulation emotions			
Curiosity		−.034	−.005
Confused		−.052	−.041
Ashamed		−.108	−.188
Frustrated		−.187*	−.187*
Proud		.012	.057
Nervous		−.040	−.067
Happy + Hopeful		.189**	.183*
Presimulation emotions			
Curiosity			−.054
Confused			.050
Ashamed			.119
Frustrated			−.013
Proud			−.113
Nervous			.018
Happy + Hopeful			.031
Simulation role			.096
PGY			−.056
Specialty			
Internal medicine			−.058
Emergency medicine			.215
Critical care			.065
OBGYN			.159
Variance components			
Simulation variance	.085	.047	.036
Team variance	.025	<.001	<.001
Individual variance	.044	.021	.012
ICC_S_	.150		
ICC_T>S_	.194		
ICC_I>T>S_	.271		

*Note*: The full model was significant, χ^2^(20) = 117.28, *p* < .001, pseudo *R* ^2^ = .385. Simulation role refers to participants role in the simulation as team member (assigned a value of 0) or team leader (assigned a value of 1). PGY refers to the post‐graduate year of the participant (i.e. training level). All specialty coefficients compare the specialty of interest (assigned a value of 1) to participants specializing in anaesthesia (assigned a value of 0). Anaesthesia was selected as it had the highest number of datapoints from all other specializations. OBGYN refers to the obstetrics and gynaecology specialty. The variance components represent the random effects of the model. Simulation variance refers to the variance estimate at the simulation level. ICC_S_ refers to the intraclass coefficient for the simulation level. Team variance refers to the variance estimate at the team level. ICC_T>S_ refers to the intraclass coefficient for the team level nested within the simulation level. Individual variance refers to the variance estimate at the individual participant level. ICC_I>T>S_ refers to the intraclass coefficient for the individual level nested within the team level and simulation level. AIC and BIC values can be found in Data S1: Material XVII. *p < .007, **p < .001.

#### Team mood predictors

A multi‐level model was conducted to determine whether emotion intensities after the simulation are predictors of perceived team mood (measured in the post‐SERQ). The results of the null, predictor‐only and full model are reported (Table 5). Post‐simulation frustration and the post‐simulation happiness and hopefulness variable were both significant predictors for perceptions of team mood. Higher scores for the happiness and hopefulness variable were associated with more positive perceptions of team mood, *b =* .18, *p* = .001, *η* ^2^ = .06, power > .99. Conversely, when ratings of post‐simulation frustration were higher, perceptions of team mood were lower, *b = −*.19, *p* = .003, *η* ^2^ = .04, power = .96.

**TABLE 5:** Multi‐level models of the effect of post‐simulation emotions on perceptions of team performance.

	Null	Null + post‐simulation emotions	Full model with covariates
Intercept	3.577**	4.286**	4.508**
Post‐simulation emotions			
Curious		−.030	−.033
Confused		−.117	−.112
Ashamed		−.138	−.179
Frustrated		−.067	−.058
Proud		−.032	−.046
Nervous		.057	.035
Happy‐Hopeful		.205**	.219*
Presimulation emotions			
Curious			−.072
Confused			.175
Ashamed			−.041
Frustrated			−.222
Proud			.052
Nervous			.087
Happy‐Hopeful			−.042
Simulation role			.140
PGY			−.094
Specialty			
Internal medicine			−.137
Emergency medicine			.255
Critical care			.044
OBGYN			.249
Variance components			
Simulation variance	.064	.027	.032
Team variance	.110	.055	.050
Individual variance	.147	.142	.123
ICC_S_	.091		
ICC_T>S_	.249		
ICC_I>T>S_	.384		

*Note*: The full model was significant, χ^2^(20) = 75.09, *p* < .001, pseudo *R* ^2^ = .333. Simulation role refers to participants role in the simulation as team member (assigned a value of 0) or team leader (assigned a value of 1). PGY refers to the post‐graduate year of the participant (i.e. training level). All specialty coefficients compare the specialty of interest (assigned a value of 1) to participants specializing in anaesthesia (assigned a value of 0). Anaesthesia was selected as it had the highest number of datapoints from all other specializations. OBGYN refers to the obstetrics and gynaecology specialty. The variance components represent the random effects of the model. Simulation variance refers to the variance estimate at the simulation level. ICC_S_ refers to the intraclass coefficient for the simulation level. Team variance refers to the variance estimate at the team level. ICC_T>S_ refers to the intraclass coefficient for the team level nested within the simulation level. Individual variance refers to the variance estimate at the individual participant level. ICC_I>T>S_ refers to the intraclass coefficient for the individual level nested within the team level and simulation level. AIC and BIC values can be found in Data S1: Material XVII. *p < .007, **p < .001.

### Debriefing responses (RQ4)

Out of 16 observations of *affective states* expressed spontaneously during the debriefing and used in the analysis, 8 observations were confusion, 4 were stress, 2 were happiness and 2 were nervousness (Table 6). When looking at emotion quadrants, no deactivating emotions (positive or negative), 2 positive activating emotions and 14 negative activating emotions were expressed during the debriefing. All 8 debriefing observations of *participants* who commented on their own performance were negative perceptions. From the 33 debriefing team performance observations, all but one were positive perceptions. For perceptions of *team mood*, 13 observations were positive perceptions and 2 were negative perceptions.

**TABLE 6:** Observations from SERQ responses and the coding results of debriefing sessions.

	Observations (*N*)	
	Debriefing	SERQ
Participants' observations		
Positive activating emotions		
Curious	0	14
Happy	2	12
Hopeful	0	15
Proud	0	12
Positive deactivating emotions		
Relieved	0	14
Negative activating emotions		
Nervous	2	6
Confused	10	3
Ashamed	0	4
Stressed	5	8
Frustrated	0	6
Negative deactivating emotions		
Hopeless	0	4
Individual performance		
Positive perceptions	0	31
Mixed perceptions	4	–
Negative perceptions	8	15
Team performance		
Positive perceptions	32	34
Mixed perceptions	5	–
Negative perceptions	1	12
Team mood		
Positive perceptions	14	38
Mixed perceptions	0	–
Negative perceptions	2	8
External observers' observations		
Individual performance		
Positive perceptions	25	
Mixed perceptions	6	
Negative perceptions	1	
Team performance		
Positive perceptions	39	
Mixed perceptions	0	
Negative perceptions	0	
Team mood		
Positive perceptions	31	
Mixed perceptions	0	
Negative perceptions	0	

*Note*: Observations for debriefing are defined as the presence or absence of a code/category for a participant within a debriefing video. Observations for emotions in the SERQ are defined as emotions rated as having an intensity of ‘moderate’ or higher in the post‐SERQ for a participant within a debriefing video. Observations for perceptions of individual performance, team performance and team mood in the SERQ are defined as ratings of 4 or higher for the respective item for positive perceptions, and 3 or lower for negative perceptions. If a participant is in more than one debriefing video, they can have one observation per debriefing video even if the observation is the same as another debriefing video they are in (e.g. a participant can have 2 observations for ‘confused’ if they participated in 2 different debriefing videos and were coded as ‘confused’ in both of the debriefings). These observations include participants who had observations in either the SERQ or the debriefing video, including those that were ultimately removed from analysis.

### Debriefing and SERQ alignment (RQ5)

**FIGURE 2:**
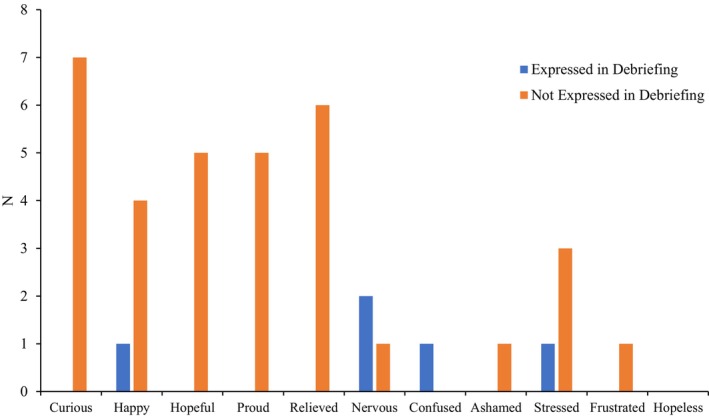
Discrete affective states reported by participants (*N*) in the SERQ as being moderately intense or higher compared with their expression during debriefing sessions.

From the observations of discrete affective states during the debriefing, 4 were in alignment with their SERQ responses while 8 were not in alignment (Table 6). However, when comparing the quadrants of emotions between the debriefing and the SERQ, 6 observations out of 14 were in alignment. When comparing affective states expressed during the debriefing to those which were reported in the SERQ as being moderate or higher in intensity, there was only 1 positive activating emotion observation (happiness) during the debriefing compared with the 22 in the SERQ, and 4 negative activating emotion debriefing observations compared with the 10 in the SERQ (Figure 2).

Alignment of perceptions for performance and team mood can be found in Table 7. Out of the 8 debriefing observations of individual performance, 4 were in alignment with their post‐SERQ perception of *individual performance* response. From the 33 observations of team performance, 24 were in alignment with their SERQ perception of *team performance* responses while 9 were not. For perceptions of *team mood*, 15 observations out of 16 were in alignment with participants' post‐SERQ responses.

**TABLE 7:** Alignment between participants' SERQ responses and debriefing responses for perceptions of individual performance, team performance and team mood.

SERQ responses	Positive perceptions in debriefing	Mixed perceptions in debriefing	Negative perceptions in debriefing
Participants' observations			
Individual performance			
Positive perceptions	0	2	4
Negative perceptions	0	2	4
Team performance			
Positive perceptions	24	3	1
Negative perceptions	8	2	0
Team mood			
Positive perceptions	13	0	0
Negative perceptions	1	0	2
External observers' observations			
Individual performance			
Positive perceptions	18	3	0
Negative perceptions	7	3	1
Team performance			
Positive perceptions	28	0	0
Negative perceptions	11	0	0
Team mood			
Positive perceptions	27	0	0
Negative perceptions	4	0	0

Chi‐square tests (Table 8) found statistically significant associations between medical trainees' responses in the SERQ and their debriefing responses for participants' perceptions of team performance (χ^2^(1) = 6.82, *p* = .009, *V* = .45, power = .97) and team mood (χ^2^(1) = 12.25, *p* < .001, *V* = .88, power > .99), specifically with the SERQ and debriefing responses being significantly aligned.

**TABLE 8:** Chi‐square tests to identify significant alignments.

	Aligned (*N*)	Not aligned (*N*)	χ^2^	Cramer's *V*
Participants' observations				
Post‐simulation emotions	6	8	.29	.14
Post‐simulation emotion quadrants	8	6	.29	.14
Perceptions of individual performance^a^	4	4	<.01	<.01
Perceptions of team performance	24	9	6.82*	.45
Perceptions of team mood	15	1	12.25**	.88
External observers' observations				
Perceptions of individual performance	19	7	5.54*	.46
Perceptions of team performance	28	11	7.41*	.44
Perceptions of team mood	27	4	17.07**	.74

^a^ Expected count < 5 for each cell in the analysis.

*p < .05, **p < .001.

## DISCUSSION

This study examined medical trainees' emotions after team‐based medical simulations and their effects on perceptions of performance and team mood while accounting for nesting effects. Multi‐level model analyses revealed differences between team leaders and members in their post‐simulation emotions, and significant effects on team performance and mood. Debriefing observations indicated potential limitations in how medical trainees discuss emotions, performance and team mood during debriefings, indicating potential gaps in current debriefing frameworks or their applications. The first evidence of validity for the SERQ is promising, indicating its potential use in future research and educational endeavours with revisions. These findings can inform training strategies for medical trainees to facilitate success in simulations and future practice.

### Medical trainees' emotions and effects on performance and mood

When comparing the self‐reported emotions of team leaders and team members, leaders reported significantly higher levels of post‐simulation shame in the SERQ. In the medical education literature, shame has emerged as an emotion of interest due to its associations with mental health and learning (e.g. Bynum et al., 2019; Nomura et al., 2021). Leaders may be more self‐critical compared with their team members due to being in the ‘hot seat’. The findings on shame align with prior research suggesting that leadership roles heighten emotional vulnerability due to increased responsibility and perceived scrutiny (Madsgaard et al., 2022). Thus, the discrepancy between leaders' and team members' shame indicates there may be a need to better support trainees as they transition into leadership roles to help them manage the increased responsibility and perceived scrutiny of individuals in their role.

None of the post‐simulation emotions significantly predicted perceived *individual* performance. However, post‐simulation happiness and hopefulness, categorized as positive activating emotions in CVT, were positively associated with perceived *team* performance. Similar findings have been reported for university students, where their task‐related emotions were associated with team rather than individual performance (Heerdink & Homan, 2024). This may suggest that post‐simulation positive activating emotions are more strongly linked to group‐level evaluations, like perceived team performance, which are often more salient in team‐based scenarios as outcomes are shared by team members. Individual performance, by comparison, may exert less influence on or be less influenced by emotions in these settings. These findings highlight that the effects of emotions are context‐dependent and shaped by the social and contextual factors.

The findings for perceived team performance align with CVT, which postulates that positive activating emotions tend to improve performance. Our findings add to prior work by highlighting the role of positive emotions in medical simulation contexts. Duffy et al. (2020) found that negative activating and deactivating emotions were negatively correlated with performance. Their study used emotion quadrants to analyse all emotions instead of discrete emotions, which may have obscured the results of individual emotions, including specific positive emotions. Our results show that discrete positive emotions also matter and warrant further study, possibly due to the approach of analysing most emotions discretely.

Next, it was found that trainees' post‐simulation frustration (negative predictor) and the post‐simulation happiness and hopefulness variable (positive predictor) were significant predictors for ratings of team mood. By contrast, other post‐simulation emotions were not significant predictors of team mood, which may reflect these emotions focus on the individual and centre on personal outcomes or internal states rather than interpersonal dynamics. As such, these emotions may be less likely to contribute to emotional contagion, aligning with prior literature suggesting that socially oriented emotions that have more outward expressions (e.g. happiness, frustration) are more likely to contribute to emotional contagion and be perceived by others in group settings (Barsade, 2002; van Kleef & Côté, 2022). It is also possible that some of the emotion predictors in the models exhibited multi‐collinearity, despite our best efforts, which contributed to the non‐significance of individual predictors despite the models overall explaining substantial variance.

The significant predictors of perceived team mood revealed that, consistent with CVT, the valence of emotions is associated with how trainees perceive their team's mood. These findings also align with and extend the current understanding of emotion transmission in educational settings (Herrando & Constantinides, 2021; Mitchell & Boyle, 2019; Yang et al., 2021), suggesting that individuals' emotional states can influence and be influenced by the emotional expressions of their teammates and influence team dynamics in team‐based medical simulations. The findings suggest that positive emotional states in one team member may contribute to an overall positive team mood, emphasizing the interpersonal and socially contagious nature of emotions in team‐based simulation contexts. These findings also suggest simulation design could incorporate training strategies to foster positive emotions and enhance team mood and performance; however, further research on the directionality of the relationship between emotions and these constructs is needed.

Although only a subset of emotion predictors emerged as statistically significant in the multi‐level models, the models overall explained a meaningful portion of variance in the outcome measures (with the majority exceeding 20.0% of the explained variance). This suggests that, despite a limited number of individual predictors reaching significance, the combination of fixed and random effects meaningfully captured how participants emotionally responded to and evaluated the simulation experience. Thus, these results accounting for the nestedness in simulations (as accounted for by the random effects) indicate that there is a non‐negligible relationship between emotions and context (e.g. individual participant, team composition, simulation scenario) in simulation‐based education. These results reinforce the importance of ecological validity in studying complex educational environments (Markauskaite et al., 2024) and the potential influence of emotions on how trainees evaluate simulation outcomes.

The multiple membership cross‐classified structure of the dataset was captured in the multi‐level models, allowing for the real‐world training environment of medical trainees to be accounted for. This approach accounted for variation across individuals, teams and simulations, reflecting the complexity of simulation‐based medical training. All multi‐level models had at least the individual level accounting for meaningful variance. For team performance and team mood, multiple levels (e.g. individual, team, simulation) explained non‐negligible variation (ICC > .05). By including all available datapoints, regardless of repetition from the same participant, we preserved the richness of the dataset and ensured that the diversity of trainees' experiences was represented in the analysis. The meaningful variance accounted for by the multi‐level models highlights the importance of considering nestedness, especially the nestedness of multiple occasions within individuals, for emotions. Previous studies that had nestedness but did not account for it within their analyses (e.g. Keskitalo & Ruokamo, 2021) could possibly have generated incomplete conclusions.

Our multi‐level approach provides a methodological advance for emotion research in medical education by enabling a more context‐sensitive and ecologically valid understanding of medical trainees' emotions regarding team‐based simulations. This matters because it allows us to capture influences from the nestedness of the data that other studies may have missed. As a result, our findings offer a more complete picture of emotions in medical education. Thus, our findings help advance the understanding of emotions and their effects in medical education towards being more accurate of real‐world medical education environments and encourage others to use similar approaches in their research.

### Medical trainees' expressions during debriefings and validity evidence

The first evidence of validity gathered for the SERQ demonstrated validity evidence based on *content*, *response processes*, *internal structure* and *relations to other variables*, particularly with post‐simulation emotions. From the debriefings, while a majority of the emotion quadrants (57%) were aligned between participants' SERQ and debriefing responses, only 43% of discrete emotions (i.e. confusion) were aligned, which may be indicative that the SERQ may not capture or accurately reflect specific emotions experienced by participants right after the simulation but may capture more general emotional states (i.e. emotion quadrants). Non‐researcher controlled debriefings may explain mismatches with SERQ data as debriefers may not have prompted participants to elaborate on the full range of emotional experiences the participants had or the participants may not have felt comfortable sharing their emotions with the medical expert debriefer due to perceived scrutiny. Other explanations for misalignment could be that the wording in the SERQ prompted a different *time frame* than the debriefing (i.e. current in the SERQ, retrospective in the debriefing), the debriefing specified tasks or outcomes (different *object foci*), debriefing responses were influenced by the trajectory or comments made by others or that participants felt more comfortable discussing certain emotions over their most intense emotions.

Though debriefing frameworks explore trainees' emotional reactions, negative activating emotions were found to be expressed the most during the debriefings despite positive activating emotions being the most reported at intensities of moderate or higher in the SERQ. This may be due to debriefings focusing on challenges or issues that occurred during the simulation to teach trainees how to navigate those particular tasks or timepoints. However, there may also be a gap in debriefing frameworks or in the training debriefers and/or trainees receive such that positive emotions are not being discussed to the degree they should be, especially if these emotions relate to critical points during the simulations. Existing debriefing models, like PEARLS (Bajaj et al., 2018), may unintentionally prioritize reflections on performance over emotional reflections. This warrants a larger focus on *all* emotions participants experience, especially positive ones, aligning with multi‐level analyses results.

It is also interesting to note the discrepancy in how many perceptions of individual performance debriefing observations there were (*n* = 8) compared with perceptions of team performance and team mood. The small number of observations limited the ability to assess alignment between individual performance debriefing observations and SERQ responses statistically. Additionally, individual performance may be discussed more critically during debriefings to identify areas of improvement, rather than what went well. With only half of the individual performance debriefing observations aligning with the SERQ responses, it is possible that the current wording of the SERQ asking participants to reflect on their *satisfaction* with their performance may be interpreted differently from how participants perceived their own performance. For example, a participant may be *satisfied* with their performance given their skill level and prior knowledge or performance in a critical task during the simulation but may not have perceived that their performance was *satisfactory* in terms of competence.

With 73% of participants' perceptions of team performance debriefing responses significantly aligned with their SERQ responses, the SERQ demonstrates promising validity evidence based on *content* and *relations to other variables* for perceptions of team performance. Perceptions of team mood were also significantly aligned between participants' debriefing responses and SERQ responses, with only one participant observation (6%) being misaligned. With such high rates of alignment, the SERQ exhibits promising validity evidence of *relation to other variables* for capturing participants' perceptions of team mood and perceptions of team performance.

Overall, the SERQ exhibited promising validity evidence based on *content* and *relations to other variables* as demonstrated by the alignment between debriefing and self‐report responses, especially for the single‐item responses for perceptions of team performance and mood. The current validity evidence for the post‐SERQ emotion items and perceptions of individual performance indicates that these items, while capturing participants' experiences to some extent, may require further revisions. Alternatively, and we believe more likely, they may indicate that the unscripted debriefings in this study alone are not sufficient to accurately obtain participants' experiences regarding simulations, with other methods, such as semi‐structured interviews or retrospective video reviews, possibly allowing for richer data sources for validity evidence. With additional sources of validity evidence, the SERQ shows promise as a research and educational tool.

### Limitations and future directions

The study was conducted in a single North American institution, which may limit the generalizability of the findings to other cultural or institutional contexts. Participants may have difficulty accurately identifying their emotions, perceptions or distinguishing between an emotion for a given subtask at a given time in the simulation scenario versus emotions over the entire set of subtasks in the simulation scenarios. Participants had to accomplish multiple subtasks throughout the simulations, and some tasks at a given time in the simulation may have been more important and more emotion‐generating than others. Participants were also asked to report their current emotional state immediately after the simulation, which did not provide participants the opportunity to report on their dynamic emotional fluctuations during the simulation. It should also be noted that simulations varied in presimulation procedures, with some allowing residents to volunteer for roles, others being preassigned but notified in advance and others finding out their role right before entering the simulation. This could have led to significant variance in the reported presimulation emotions and therefore affected the models.

Methodologically, one limitation was power constraints limiting our ability to include all emotions and covariates in our statistical analysis. Despite these constraints, we conducted multi‐level modelling with all emotions and covariates and discovered similar results (Data S1: Material XVIII–XX). We also used listwise deletion to handle missing data, which may have introduced bias if the data were not truly missing completely at random and may have affected our findings. Furthermore, Likert‐scale items were treated as continuous. However, this approach may not fully reflect the ordinal nature of the data, which could bias results. Future studies should explore methods that reflect the ordinal nature of self‐report data.

The validity evidence presented also draws into question the strength of our findings as it is possible the SERQ does not adequately capture perceptions of concurrent emotions after the simulation or individual performance. However, the validity evidence was limited by non‐researcher controlled or led debriefings and a lack of probing. Expert debriefers largely did not directly probe participants regarding the extent of post‐simulation emotions or tease apart perceptions of individual performance from team performance. Participants were also not asked about their teammates' feelings, which may have limited elaboration, affecting alignment with their SERQ responses.

Future research should use multi‐level models, especially when in hierarchical or team‐based situations. Further research should examine additional measures of emotion to help overcome the limitations of self‐report measures, including those with good evidence of validity (Duffy et al., 2020). Additional validity evidence should be collected, ideally including other data channels (e.g. semi‐structured interviews). As effective emotion regulation can improve team members' and leaders' perceptions of team mood and performance, medical trainees' emotion regulation strategies should also be examined to determine their effectiveness and impact on performance.

## CONCLUSIONS

Our multi‐level analyses revealed the importance of accounting for nesting within team‐based simulations, specifically at the individual, team composition and simulation scenario levels, potentially challenging the strength of previous related work. Our results provide insight into potential areas to expand medical training, specifically regarding which emotions to target for regulation to possibly improve perceptions of team mood and team performance. Importantly, our work lays the foundation for future medical education multi‐level analyses on emotions and related constructs, which may lead to adjustments in the medical curricula research and also provides evidence that team members' self‐reported emotions are at least as important to consider in research and simulation training as team leaders'.

Additionally, the items for emotions, perceptions of team performance and team mood in the SERQ have demonstrated promising validity evidence within team‐based medical simulations. With the SERQ's narrative‐style summary sent directly to participants and its potential to serve as a reflective tool, the SERQ may be used in the future as a research and educational tool, especially as efforts are underway to provide further validity evidence from additional data channels and participant populations (i.e. interprofessional medical teams).

## AUTHOR CONTRIBUTIONS

**Keerat Grewal:** Conceptualization; software; data curation; formal analysis; investigation; funding acquisition; methodology; project administration; visualization; writing – original draft. **Sayed Azher:** Software; methodology; investigation; writing – review and editing. **Matthew Moreno:** Methodology; investigation; project administration; writing – review and editing. **Reinhard Pekrun:** Funding acquisition; methodology; writing – review and editing. **Jeffrey Wiseman:** Funding acquisition; writing – review and editing; methodology. **Jessica Flake:** Formal analysis; writing – review and editing; resources. **Susanne Lajoie:** Funding acquisition; methodology; writing – review and editing. **Ning‐Zi Sun:** Methodology; resources; writing – review and editing. **Gerald M. Fried:** Funding acquisition; writing – review and editing; methodology. **Elene Khalil:** Methodology; resources; writing – review and editing. **Jason M. Harley:** Conceptualization; funding acquisition; methodology; project administration; resources; supervision; formal analysis; investigation; writing – review and editing.

## FUNDING INFORMATION

This work was supported by a Partnership Development Grant from the Social Sciences and Humanities Research Council of Canada [grant number: 890‐2020‐0060] awarded to the senior author and P.I., J.M. Harley. This work was also supported by a doctoral research scholarship from the Fonds de recherche du Québec—Société et culture (330021) and the Social Sciences and Humanities Research Council of Canada (752–2024‐1350) awarded to the first author, K. Grewal.

## CONFLICT OF INTEREST STATEMENT

The authors report no conflicts of interest.

## CONSENT

Prior to data collection, participants provided informed consent and were informed of their right to freely withdraw consent at any time during the data collection. Consent was obtained via a signature and written approval on a consent form approved by our institutional research ethics board.

## Supporting information


Data S1.

## Data Availability

The participants of this study did not consent to having their data shared publicly and are from a small and highly specialized professional population. As such, due to privacy concerns, data will not be made available.

## APPENDIX A Demographic information of participants

**Table:**

Demographic	*N* (obs)	Mean ± SD (range)
**Age, years**		29.2 **±** 3.6 (24–38)
**Gender**		
Female	38 (83)	
Male	47 (90)	
**Ethnicity**		
Arab	8 (19)	
Black	2 (6)	
Caucasian	57 (106)	
Chinese	10 (18)	
Indigenous	1 (4)	
Japanese	1 (1)	
Korean	2 (8)	
South Asian	2 (5)	
Southeast Asian	2 (2)	
West Asian	1 (3)	
Other	1 (1)	
**Resident level**		3.0 **±** 1.8 (1–8)
PGY 1	25 (42)	
PGY 2	21 (38)	
PGY 3	15 (40)	
PGY 4	7 (13)	
PGY 5	10 (19)	
PGY 6	7 (17)	
PGY 7	1 (1)	
PGY 8	1 (3)	
**Department**		
Emergency department	22 (50)	
Medical or surgical wards	34 (51)	
Operating poom	21 (48)	
Post‐anaesthesia care unit	2 (3)	
Medical or surgical clinics	2 (2)	
Other	10 (19)	

## APPENDIX C Coding scheme used for post‐simulation debriefing responses

**Table:**

Superordinate category	Definition	Subordinate category 1	Definition	Subordinate category 2	Definition	Example
Post‐simulation emotion	An emotion that was experienced either after the simulation was completed, or that was experienced during the simulation but remained unresolved after the completion of the simulation. Participants either explicitly state the emotion experienced (e.g. stressed) or indirectly state the emotion experienced, either through comments or questions (e.g. asking many questions about elements of the case).	Positive Activating Emotion	An emotion that is positively valenced and activates physiological arousal. Participants express positive sentiments about the simulation, either explicitly or indirectly, and demonstrate engagement or heightened alertness or arousal.	Curious	A feeling of intrigue or curiosity, or an expression of wanting to acquire knowledge	
				Happy	A feeling of great pleasure and joy	‘I think it was a very positive experience’. (PN8. T2A)
				Hopeful	A feeling of optimism or desire for a positive outcome or success when the outcome is uncertain	
				Proud	A feeling of elation and esteem of one's achievement where success is attributed to one's abilities or effort	
				Other	An emotion that is positively valenced and activates physiological arousal, but is not any of the other positive activating discrete emotions	
		Positive deactivating emotion	An emotion that is positively valenced and does not activate physiological arousal. Participants express positive sentiments about the simulation, either explicitly or indirectly, and demonstrate little to no engagement or reduced alertness or arousal.	Relief	A feeling of ease as a distressing condition ceases or changes for the better	
				Other	An emotion that is positively valenced and does not activate physiological arousal, but is not relief	
		Negative Activating Emotion	An emotion that is negatively valenced and activates physiological arousal. Participants express negative sentiments about the simulation, either explicitly or indirectly, and demonstrate engagement or heightened alertness or arousal.	Confused	A feeling of uncertainty, or a lack of understanding	‘I felt like I wasn't sure 100% like what was going on in one quarter versus another’. (PN2, T1B)
				Ashamed	A feeling of disgrace, humiliation, dissatisfaction or deflation related to an awareness of a personal failure or shortcoming that does not meet perceived standards or due to discordance between an alternate outcome and the actual outcome	
		
				Stressed	A feeling of worry or heightened awareness about a possible negative outcome, failure when the outcome is uncertain, or elements in the simulation threatening the success of the case	‘It felt quite overwhelming, I felt like there was a lot of stuff going on at once’ (PN2, T1B)
				Frustrated	A feeling of irritation or annoyance	
				Nervous	A feeling of worry or nervousness about a possible negative outcome or failure when the outcome is uncertain	‘And then I was like I feel more nervous after [the simulation]’ (PN1, T1B)
			Other	An emotion that is negatively valenced and activates physiological arousal, but is not any of the other negative activating discrete emotions	
		Negative deactivating emotion	An emotion that is negatively valenced and does not activate physiological arousal. Participants express negative sentiments about the simulation, either explicitly or indirectly, and demonstrate little to no engagement or reduced alertness or arousal.	Hopeless	A feeling of pessimism or feeling like a positive outcome or success is out of reach or unlikely.	
Other	An emotion that is negatively valenced and does not activate physiological arousal, but is not hopelessness			
Individual performance perception	A participant's perception of their own performance in their role in the simulation in terms of success. Participants either explicitly state how they perceive their own performance (e.g. ‘ ’) or indirectly state their perception of their own performance (e.g. ‘ ’)	Positive remarks	Participants perceive their performance as having been good or better. Comments are largely focussed on elements that went well (>75% of the comment is positive).			‘[about themself] I think the other thing that went well was like trying to summarize.’ (PN1, T1A)
		Negative remarks	Participants perceive their performance as having been ok or worse. Comments are largely focussed on areas to improve in or areas where they are lacking knowledge (>75% of the comment is negative)			‘I could've probably done a better job letting you know what was going on’. (PN4, T1B)
		Mixed remarks	Participants are not clear in their perception of performance. They may contradict themselves (comments are both negative and positive, or neutral).			‘I didn't always […] make sure people looped back to me […] but it was quiet so you could see and hear what people were doing’. (PN1, T1A)
Team Performance Perception	A participant's perception of their team's performance in the simulation in terms of success. Participants either explicitly state how they perceive their team's performance (e.g. ‘ ’) or indirectly state their perception of their team's performance (e.g. ‘ ’). Comments can be about their entire team, or about specific team members.	Positive remarks	Participants perceive their team's performance as having been good or better. Comments are largely focussed on elements that went well (>75% of the comment is positive).			‘I think it was also helpful that like we were a team. That like, we would be kind of like all agree like “should we just work together from the get‐go?”’(PN52, T18)
		Negative Remarks	Participants perceive their team's performance as having been ok or worse. Comments are largely focused on areas to improve in (>75% of the comment is negative).			‘I think we should have started the epi maybe a bit before’. (PN9, T30)
		Mixed Remarks	Participants are not clear in their perception of their team's performance. They may contradict themselves (comments are both negative and positive, or neutral)			‘I think overall the communication was decent but also could have been improved’. (PN52, T18)
Team Mood Perception	A participant's perception of their team's mood or teamwork. Participants either explicitly state how they perceive their team's mood (e.g. ‘ ’) or indirectly state their perception of their team's mood (e.g. ‘ ’)	Positive remarks	Participants perceive their team's mood as having been good or better. Comments are largely positive regarding the team and teamwork (>75% of the comment is positive)			‘Everyone is calm and orderly’. (PN6, T2B)
		Negative remarks	Participants perceive their team's mood as having been ok or worse. Comments are largely focussed on points in the simulation where there was friction and other conflicts within the team (>75% of the comment is negative)			‘I think it was a lot more difficult than the last scenario, but I think we all probably felt that it was a little more nervous in the room’. (PN1, T1B)
		Mixed remarks	Participants are not clear in their perception of their team's performance. They may contradict themselves (comments are both negative and positive, or neutral)			

*Note*: Categories without examples are due to there being no data corresponding to that particular code.

## APPENDIX D Null multi‐level models of the effect of simulation role on post‐simulation emotions

**Table:**

	Curiosity	Confused	Ashamed	Frustrated	Proud	Nervous	Happy and hopeful component
Intercept	2.930**	2.333**	1.814**	1.948**	2.687**	2.244**	.086
Variance components							
Simulation variance	–	.281	.187	–	–	.108	–
Team variance	–	–	–	.090	–	–	–
Individual variance	.114	.151	.262	.259	.339	.390	.722
ICC_S_	–	.264	.202	–	–	.130	–
ICC_T_	–	–	–	.083	–	–	–
ICC_I_	.098	.406	.485	.323	.352	.599	.401

*Note*: Presented are the final null models with levels with low intraclass coefficient values removed (ICC < .05). The variance components represent the random effects of the model. Simulation variance refers to the variance estimate at the simulation level. ICC_S_ refers to the intraclass coefficient for the simulation level. Team variance refers to the variance estimate at the team level. ICC_T_ refers to the intraclass coefficient for the team level. Individual variance refers to the variance estimate at the individual participant level. ICC_I_ refers to the intraclass coefficient for the individual level. All ICC values are calculated to account for the cross‐classification of higher levels in the model (i.e. ICC_I_ is calculated based on the variance at the individual level and the variance at higher levels within the model).***p* < .001.

## APPENDIX E Predictor‐only multi‐level models of the effect of simulation role on post‐simulation emotions

**Table:**

	Curiosity	Confused	Ashamed	Frustrated	Proud	Nervous	Happy and hopeful component
Intercept	2.987**	2.295**	1.658**	1.821**	2.678**	2.192**	.101
Simulation role	−.179	.108	.494**	.379	.028	.163	−.046
Variance components							
Simulation variance	–	.274	.237	–	–	.108	–
Team variance	–	–	–	.106	–	–	–
Individual variance	.112	.152	.275	.307	.338	.379	.724

*Note*: Simulation role refers to participants role in the simulation as team member (assigned a value of 0) or team leader (assigned a value of 1). The variance components represent the random effects of the model. Simulation variance refers to the variance estimate at the simulation level. Team variance refers to the variance estimate at the team level. Individual variance refers to the variance estimate at the individual participant level. ***p* < .001.
