# Supplementary material for: Medical trainees' emotions and their effects on perceptions of performance and team mood in team‐based simulations
Source: Br J Educ Psychol. 2025 Aug 13;96(1):306–36. doi: 10.1111/bjep.70017 (PMC12879525; doi:10.1111/bjep.70017)
Supplement: Supplementary file 1 — Data S1. [file BJEP-96-306-s001.docx]

**Supplemental Material I**

*Justification for Emotion Scale in SERQ.*

The SERQ was designed to capture participants’ concurrent emotions before and after a simulation, as well as perceptions regarding performance and team mood. Using *The Standards for Educational and Psychological Testing* (American Educational Research Association et al., 2014) as the guiding framework for collecting validity evidence, we focused primarily on evidence based on *content*, *internal structure*, *response processes*, and *relations to other variables* for the SERQ. Evidence based on *content* refers to the relevance and representativeness of the underlying constructs of a measure to a given population of participants. Evidence based on *internal structure* refers to the items within a measure demonstrating relationships expected based upon conceptual frameworks. Evidence based on *response processes* pertains to the cognitive processes that the participants engage in when using a measure, including how participants interpret and understand items within the measure. Evidence based on *relations to other variables* compares variables within a measure to either other variables within the measure or to external measures (i.e., external validity) to establish convergence or divergence for similar constructs.

Theoretical and previous empirical evidence was used to develop the SERQ to ensure it was relevant and representative for the participants, providing validity evidence based on *content*. Specifically, the SERQ’s development was informed by theoretical models of achievement emotions (Harley et al., 2019; Harley & Pekrun, 2024; Pekrun, 2006, 2024), and drew from existing self-report instruments with evidence of validity, most notably the Medical Emotion Scale (MES; Duffy et al., 2020), which has demonstrated validity evidence with single-item emotional state measures in medical education contexts. Like the MES, we also collected data on concurrent emotional states from one moment in time, reducing recall effects as participants were not asked to recall their emotional states from the simulation. We selected emotions that spanned the four quadrants of emotions (positive activating, positive deactivating, negative activating, and negative deactivating) and were relevant to team-based simulation contexts. Curiosity and confusion were included as epistemic emotions relevant to learning and information processing (Vogl et al., 2020); shame, frustration, hopelessness, and nervousness as negative emotions likely to arise in high-pressure achievement situations (Pekrun & Stephens, 2010); pride, hopefulness, and happiness as positive achievement emotions linked to success and motivation (Frumos et al., 2024); and relief as a common response following the cessation of an unpleasant situation (Graham et al., 2023). Stress was also included given its practical relevance in medical education (Ahn et al., 2023; LeBlanc & Posner, 2022).

The same set of emotion items was used in both the pre- and post-SERQs to allow for consistency across timepoints and to reflect that all of the included emotions can be anticipatory and retrospective. For example, pride–which is typically retrospective–may be felt in anticipation of performing well before a simulation, shame may arise out of fear of poor upcoming performance, and hopelessness may reflect a lack of confidence about a future simulation. After a simulation, these emotions are likely to be retrospective: pride may be experienced as a reflection of a successful performance, shame may arise in response to a perceived mistake, and hopelessness may be experienced if the simulation that has concluded was perceived as overwhelming or unsuccessful. Using the same set of emotion items across both timepoints enabled consistent measurement across timepoints, recognizing that the object focus of each emotion may vary depending on individual appraisal and context.

As the SERQ was to be administered to medical trainees who may or may not have previous exposure to concepts of emotion or emotion regulation, it was imperative that the terminology used was easy to understand and interpretable by a general audience. Though stress is typically described as an affective state rather than an emotion (Gross, 2024), it is also used interchangeably with anxiety and nervousness, both colloquially and in the literature (Ahn et al., 2023; Gross, 2024; LeBlanc & Posner, 2022). Objective stress, referring to physiological responses (e.g., decreased heart rate variability), and subjective stress, referring to personal appraisals and perceived stress, can relate to various stressors, such as task difficulty (Joseph et al., 2022). As such, stress was included in the SERQ as well as nervousness to examine the relationship between these states in the medical education context (analyses with stress and nervousness can be found in Supplemental Materials VI-VIII, XVI-XVIII), though it was not formally analyzed in this study since we focused on emotions. The addition of nervousness and stress is consistent with other scales used to measure emotions in several studies, such as the Positive and Negative Affect Schedule (PANAS; Watson et al., 1988) which includes “nervous” and the Socio-Emotional Sampling Tool (Bakhtiar et al., 2018) which includes “stressed”.

Given the need for brevity during simulation training, we opted for single-item emotion indicators, as used in the MES and other similar single-item scales with validity evidence, such as the Emotion-Value Questionnaire (Harley et al., 2015) and Epistemic Emotion Scale (Pekrun et al., 2017). Additionally, Gogol and colleagues (2014) found that single-item scales are viable to use when time is limited and are psychometrically sound compared to multi-item affective scales. The wording of SERQ emotion items closely mirrors the MES and items from other tools, such as the Achievement Emotions Questionnaire (Pekrun et al., 2011). Furthermore, previous work has provided evidence of validity based on *response processes* (i.e., evidence of how participants interpret and understand items) by demonstrating that the adjective rating scale is understood and interpreted similarly by individuals (e.g., Duffy et al., 2020; Fontaine et al., 2013; Shuman & Scherer, 2013).

Research collaborators with expertise in emotion, educational psychology, medicine, and medical education provided iterative feedback during meetings throughout the development of the SERQ. Initial meetings involved reviewing existing instruments and identifying appropriate tools and constructs to adapt for the SERQ. In subsequent sessions, collaborators were presented with draft versions of the SERQ items, engaged in structured group discussions, and provided detailed feedback on item inclusion, wording, relevance, and how to best preserve the theoretical and psychometric properties of the adapted items to support validity evidence based on *content* and *internal structure*. Collaborators also trialed the SERQ and offered feedback based on their user experience and expertise. The feedback from the research collaborators helped to ensure any modifications made to the SERQ were grounded in theoretical, empirical, or practical considerations.

Practical considerations for the SERQ included ensuring: 1) its brevity to prevent survey fatigue and fit within the logistical constraints of simulation training days, 2) survey items were understandable and relevant to participants, and 3) survey items were comprehensive enough to cover the expected range of responses and experiences. As an example, boredom was not included in the SERQ as it was not an emotion that would be expected within the crisis scenarios of the simulations. Emotion labels were selected to represent clusters of theoretically or practically similar emotions, such as happiness encompassing enjoyment (Lyubomirsky & Kurtz, 2009).

Prior to the use of the SERQ in the current study, informal semi-structured interviews were conducted with four medical experts external to the research team. These individuals reviewed the SERQ for clarity, relevance, feasibility, and comprehensiveness, and their feedback informed final revisions of the SERQ. As such, these interviews provided validity evidence based on *content* as enhancements to the relevance, representativeness, and clarity of the SERQ were made based on the feedback received.

Bivariate correlation strengths were defined according to Dancey and Reidy (2007). Correlations between emotions (Supplemental Material VII-VIII) demonstrated that emotions sharing a common position in a valence and activation quadrant (e.g., negative activating emotions) typically exhibited moderate (defined as .40 ≤ *r* < .70) to strong (defined as .70 ≤ *r* < 1.00) positive correlations. Conversely, emotions that did not share positions in a common valence and activation quadrant exhibited negligible (defined as .00 ≤ *r* < .10) to weak (defined as .10 ≤ *r* < .40) positive correlations or were negatively correlated. These findings were consistent with theoretical (e.g., Pekrun, 2006, 2024) and empirical (e.g., Duffy et al., 2020) expectations, providing further evidence of validity based on *internal structure* and *content.*

Though the SERQ also contains items related to emotion regulation, perceptions of emotion regulation effectiveness, and additional effects of emotion regulation strategies on the simulation, these items were not analyzed in the current study due to the scope of our research questions.

**Supplemental Material II**

*Additional Details Regarding the Use of Listwise Deletion*

Listwise deletion was used to maintain consistency across models; however, we acknowledge this approach may introduce bias if the data are not missing completely at random (Li, 2013). Multiple imputation was not used as it would require taking into account the complexity of the cross-classified, nested nature of the dataset (Wijesuriya et al., 2022), which, if done incorrectly, could introduce bias and undermine the integrity of the model estimates. The missing data arose from the variables that were used as covariates in the models, meaning that participants’ data were excluded when they did not provide data on their training level and/or specialty, and/or did not complete the pre-SERQ and thus did not provide pre-simulation emotion data. Little’s test (Little, 1988) was used to test the assumption of data missing completely at random, and found that the data does not violate the assumption, χ^2^(14)=22.28, *p*=.073.

**Supplemental Material III**

*Development of the SERQ.*

To the authors’ knowledge, there is no self-report tool that simultaneously captures emotional states, perceptions of performance, and team mood in medical education settings. Existing self-report tools, such as the Achievement Emotions Questionnaire (Pekrun et al., 2011, 2023) or the Ottawa Crisis Resource Management Global Rating Scale (Kim et al., 2006), are limited to collecting data on a few specific constructs (e.g., emotions or performance) in isolation. Researchers often require multiple tools to capture these constructs, which may be infeasible (e.g., due to time constraints) or impractical (e.g., due to survey fatigue) in applied research settings, such as simulation training (Colbert et al., 2021; Thibault et al., 2022).

The SERQ was envisioned to allow (1) researchers to collect self-report data from medical trainees on their concurrent emotional states before and after simulations, and their perceptions of performance, team mood, and regulation strategies during simulations, (2) educators the ability to use this information to help guide debriefing content, especially during the emotional reactions phase, and (3) trainees the opportunity to reflect on their experiences before, during, and after simulations to potentially discuss during debriefings. Wording in the SERQ deliberately did not use jargon (e.g., emotion quadrants), and specialty (e.g., internal medicine) or profession-specific language (e.g., lead physician).

**Supplemental Material IV**

*Description of Post-Simulation SERQ Used in Analysis*

[Question] Please indicate how you feel **right now** about the **simulation** you have just completed. [single-item response for multiple items]

[Items] curious, confused, ashamed, relieved, stressed, frustrated, hopeless, happy, hopeful, proud, nervous

[Response options; radio buttons] not at all, very little, moderately, strongly, very strongly

[Question] *When I think about the simulation I have just completed, the overall mood in my group was _______* [single-item response]

[Response options; drop-down list] very positive, positive, neutral, negative, very negative

[Question] *Overall, I am ________ satisfied with how I performed today.* [single-item response]

[Response options; drop-down list] extremely, very, moderately, not very, not at all

[Question] *Overall, I am ________ satisfied with how my group performed today.* [single-item response]

[Response options; drop-down list] extremely, very, moderately, not very, not at all

**Supplemental Material V**

*Justification for Items Used in Analyses.*

Initially, exploratory factor analysis was conducted to determine if emotions were loading on factors. However, the factor analysis revealed that several emotions were cross-loading onto multiple factors (Supplemental Material VI) (Taber, 2018). As such, we decided to analyze emotions discretely instead of using a quadrant (valence-activation) model. Analyzing emotions discretely would also provide the advantage of being able to better interpret results as the results are more specific for each emotion. However, the number of items included in our analysis needed to be reduced to address issues of multicollinearity and power issues.

To improve interpretability and reduce conceptual overlap, affective states that were not emotions were removed, resulting in the removal of stress (Ahn et al., 2023; Gross, 2024; Jarrell & Lajoie, 2017). Then, emotions with low mean levels (less than 2 -“very little” for both leaders and team members) indicating they were experienced at low levels were removed, resulting in the removal of hopelessness.

Next, the correlations of the remaining emotions were reviewed (Supplemental Material VII-VIII). Emotions showing strong intercorrelations (i.e., happiness and hopefulness) underwent a principal component analysis (PCA) to determine whether conceptually similar emotions could be combined to reduce the number of predictors in the multilevel models, thereby addressing power limitations. This resulted in happiness and hopefulness being combined into one variable (Supplemental Material IX). Rather than selecting only one of these two emotions, we chose to combine them because both were considered educationally relevant and important in achievement settings such as simulation-based training. Combining them preserved meaningful variance from both constructs, while reducing the number of predictors and helping to avoid issues of multicollinearity and underpowered analyses. Finally, the evaluation of the education relevance of the remaining emotions resulted in the removal of relief. All other emotions were analyzed discretely.

**Supplemental Material VI**

*Exploratory factor analysis with Orthogonal Varimax Rotation of Post-Simulation Emotions.*

| ***Variable*** | ***Loadings*** | | | |
| --- | --- | --- | --- | --- |
|  | **Factor 1** | **Factor 2** | **Factor 3** | **Factor 4** |
| Post-Curious | 0.106 | *0.380* | *0.394* | -0.133 |
| Post-Confused | **0.568** | -0.147 | *0.389* | 0.115 |
| Post-Ashamed | **0.744** | -0.010 | 0.122 | 0.197 |
| Post-Relieved | 0.139 | **0.608** | 0.037 | -0.131 |
| Post-Stressed | **0.849** | 0.011 | 0.008 | -0.107 |
| Post-Frustrated | **0.736** | -0.132 | -0.025 | 0.224 |
| Post-Hopeless | **0.632** | -0.030 | -0.021 | 0.319 |
| Post-Happy | -0.069 | **0.806** | 0.109 | -0.011 |
| Post-Hopeful | 0.016 | **0.783** | -0.016 | 0.043 |
| Post-Proud | -0.153 | **0.747** | -0.098 | -0.053 |
| Post-Nervous | **0.783** | 0.021 | 0.064 | -0.208 |
| Eigenvalue | 3.21 | 2.37 | 0.35 | 0.30 |
| % of total variance | 58.76 | 43.47 | 6.40 | 5.45 |

*Note*. Bolded values indicate primary loadings for each post-simulation emotion. Italicized values indicate the presence of cross-loading for the post-simulation emotion.

**Supplemental Material VII**

*Bivariate Pearson Correlations of Independent Variables.*

|  | 1 | 2 | 3 | 4 | 5 | 6 | 7 | 8 | 9 | 10 | 11 | 12 | 13 |
| --- | --- | --- | --- | --- | --- | --- | --- | --- | --- | --- | --- | --- | --- |
| 1 Perceived individual performance | - |  |  |  |  |  |  |  |  |  |  |  |  |
| 2 Perceived team performance | .53** | - |  |  |  |  |  |  |  |  |  |  |  |
| 3 Perceived team mood | .57** | .52** | - |  |  |  |  |  |  |  |  |  |  |
| 4 Curiosity | .22* | .15 | .15 | - |  |  |  |  |  |  |  |  |  |
| 5 Confusion | -.51** | -.32** | -.45** | .02 | - |  |  |  |  |  |  |  |  |
| 6 Shame | -.44** | -.25* | -.42** | .02 | .58** | - |  |  |  |  |  |  |  |
| 7 Relief | .26* | .21 | .28** | .34** | -.22* | -.13 | - |  |  |  |  |  |  |
| 8 Stress | -.37** | -.25* | -.48** | -.07 | .35** | .51** | -.14 | - |  |  |  |  |  |
| 9 Frustration | -.50** | -.35** | -.54** | -.30** | .50** | .52** | -.16 | .48** | - |  |  |  |  |
| 10 Hopelessness | -.39** | -.36** | -.43** | -.11 | .44** | .55** | -.16 | .42** | .59** | - |  |  |  |
| 11 Happiness | .51** | .39** | .56** | .37** | -.25* | -.20 | .47** | -.32** | -.39** | -.35** | - |  |  |
| 12 Hopefulness | .34** | .19 | .38** | .27* | -.24* | -.19 | .38** | -.37** | -.32** | -.26* | .73** | - |  |
| 13 Pride | .58** | .20 | .36** | .20 | -.37** | -.47** | .37** | -.39** | -.39** | -.31** | .62** | .56** | - |
| 14 Nervousness | -.46** | -.33** | -.52** | -.09 | .39** | .42** | -.06 | .68** | .46** | .29** | -.34** | -.21 | -.36** |

*Note.* **p* < 0.05, ***p* < 0.01. Correlations were conducted with only the first data point of participants in the data set to avoid violations of the assumption of independence.

**Supplemental Material VIII**

*Bivariate Pearson Correlations of Pre-Simulation Emotions.*

|  | 1 | 2 | 3 | 4 | 5 | 6 | 7 | 8 | 9 | 10 | 11 |
| --- | --- | --- | --- | --- | --- | --- | --- | --- | --- | --- | --- |
| 1 Curiosity | - |  |  |  |  |  |  |  |  |  |  |
| 2 Confusion | .12 | - |  |  |  |  |  |  |  |  |  |
| 3 Shame | .02 | .51** | - |  |  |  |  |  |  |  |  |
| 4 Relief | .23* | -.10 | -.10 | - |  |  |  |  |  |  |  |
| 5 Stress | .08 | .39** | .35** | -.31* | - |  |  |  |  |  |  |
| 6 Frustration | -.18 | .34** | .35** | -.05 | .22 | - |  |  |  |  |  |
| 7 Hopelessness | -.04 | .45** | .46** | -.11 | .27* | .42** | - |  |  |  |  |
| 8 Happiness | .43** | -.19 | -.19 | .41** | -.41** | -.11 | -.30* | - |  |  |  |
| 9 Hopefulness | .56** | -.02 | -.13 | .49** | -.16 | -.03 | -.22 | .55** | - |  |  |
| 10 Pride | .34** | -.01 | .01 | .65** | -.18 | .08 | -.22 | .46** | .55** | - |  |
| 11 Nervousness | .32** | .25* | .32** | -.30** | .74** | .17 | .21 | -.24* | .02 | -.09 | - |

*Note.* **p* < 0.05, ***p* < 0.01. Correlations were conducted with only the first data point of participants in the data set to avoid violations of the assumption of independence.

**Supplemental Material IX**

*Principal Component Analysis of Post-Simulation Happiness and Hopefulness.*

|  | ***Comp1*** | ***Comp2*** |
| --- | --- | --- |
| Post-Happy | 0.7071 | 0.7071 |
| Post-Hopeful | 0.7071 | -0.7071 |
| % of total variance explained | 0.8478 | 0.1522 |

**Supplemental Material X**

*Coding of External Observers and Debriefers – Source of Validity*

There were a total of 9 external observers (i.e., medical experts leading the debriefing, peers observing the simulations). When performance comments were directed at specific participants, these were considered external perspectives of individual performance. When performance comments included verbiage to indicate they were applicable to all members of a team, they were considered external perspectives of individual and team performance. When there was no indication of a performance comment being directed to a specific participant, the comment was considered an external perspective of team performance.

For the debriefing responses that were performance-related or team mood-related, the codes for each external observer were used to categorize them into “positive perceptions”, “negative perceptions”, and “mixed perceptions” for team mood, team performance, and individual performance. “Positive perceptions” had greater than 50% of their responses be positively-oriented, whereas “negative perceptions” were those with greater than 50% of their responses negatively-oriented. “Mixed perceptions” had mixed responses, and included those with 50% or less of their responses categorized as negatively- or positively-oriented.

Comments regarding performance or team mood by observers or debriefing faculty (i.e., external observers) were considered external sources of validity evidence, falling under *relations to other variables*. Out of 26 observations from *observers* who commented on participants individual performance during the debriefing (25 positive perceptions; 1 negative perception), 19 were in alignment with the participant’s post-SERQ perception of *individual performance* response. From the 39 observations from observers who commented on the team’s performance as a whole (all perceptions were positive), 28 were in alignment with participants’ SERQ perceptions of *team performance* responses while 11 were not. For perceptions of *team mood* by external observers (all perceptions were positive), 27 observations out of 31 were in alignment with participants’ post-SERQ *team mood* responses.

The alignment with external observers’ perceptions of *individual performance* (χ^2^(1) = 5.54, *p* = .019, *V* = .46, power = .92)*, team performance* (χ^2^(1) = 7.41, *p* = .006, *V* = .44, power = .98)*,* and *team mood* (χ^2^(1) = 17.07, *p* < .001, *V* = .74, power > .99) were all found to have a statistically significant distribution. Specifically, the chi-square indicated that responses from external observers during the debriefing were significantly aligned with medical residents’ perceptions in the SERQ.

External observers had 26 observations for perceptions of participants’ individual performance, and all observations except one were positive perceptions of residents’ individual performance. With the external observers exhibiting significant alignment with participants’ self-reported individual performance, this further supports the notion that participants may be satisfied with their performance for their training level, but, unlike external observers, may not perceive their performance as being satisfactory. Participants may be more self-critical during the debriefing compared to external observers or may not want to express their thoughts regarding their performance, possibly due to social desirability bias, not wanting to be contradicted by the medical expert debriefer, or seeking out feedback to improve their abilities. As one of the goals of debriefing is to provide feedback and identify next steps for trainees (Sawyer et al., 2016), these findings are unsurprising, though they could serve as a barrier to trainees expressing the full extent of their thought or emotions during debriefings which is a key element of the debriefing process (Eppich & Cheng, 2015; Ross, 2021). External observers’ perceptions of team performance and team mood aligning with participants' SERQ responses also provides evidence of validity.

**Supplemental Material XI**

*Establishment of Alignment Between Debriefing Codes and SERQ Responses.*

For the alignment with the emotion scale in the SERQ, participants’ responses were considered in alignment if, during the debriefing, an emotion they expressed as having experienced during or after the simulation (but had not been resolved during the simulation) was one of the emotions they rated as being moderate or more intense in the post-SERQ. Additionally, the discrete emotions from the debriefing were categorized according to their emotional quadrant which was then compared to a participant’s top 3 emotional quadrants from their SERQ. The top 3 emotional quadrants were based upon the emotions they rated as being moderate or more intense in the post-SERQ, and tiebreaks were established by selecting the quadrant with a higher average intensity across all emotions within that quadrant. For example, if a tie-break was needed to decide whether the positive activating (encompassing curiosity, happiness, hopefulness and pride) or negative activating (encompassing confusion, shame, stress, frustration, and nervousness) quadrant would be in the top 3, the quadrant with a higher mean intensity was selected. As participants rated the intensity of all 11 emotions in the SERQ, there was potential for all emotions across all 4 emotional quadrants to be rated as moderate or higher. Thus, including the top 4 emotional quadrants may artificially inflate the congruence between the SERQ and debriefing responses as it could enable some participants to have all 4 emotional quadrants included as part of the alignment process, which would result in a 100% alignment regardless of the emotion(s) discussed during the debriefing. Alignment was considered achieved if at least one emotion discussed during the debriefing was part of an emotional quadrant that was one of the participants’ top 3 emotional quadrants in the SERQ. For example, if a participants’ top 3 emotional quadrants were negative activating emotions, positive activating emotions, and positive deactivating emotions, then if they expressed feeling confused (a negative activating emotion) during the debriefing, they were considered in alignment between their SERQ and debriefing responses.

Alignment for the performance-related and team mood-related debriefing responses with the SERQ responses was conducted. Alignment with “positive perceptions” categories was established if participants rated the corresponding item in the SERQ as a 4 or higher (SERQ performance-related responses: “satisfied”, “very satisfied”; SERQ team mood-related responses: “positive”, “very positive”). Alignment for “negative perceptions” categories was established if participants rated the corresponding item in the SERQ as a 3 or lower. “Mixed perception” categories were not used to establish alignment as they were unclear in how participants’ viewed themselves or their team in that category.

**Supplemental Material XII**

*Variance Inflation Factor Matrix of Variables Used in Emotion Multilevel Model Analyses.*

|  | ***Curiosity*** | ***Confused*** | ***Ashamed*** | ***Frustrated*** | ***Proud*** | ***Nervous*** | ***Happy and Hopeful*** |
| --- | --- | --- | --- | --- | --- | --- | --- |
| Simulation role | 1.05 | 1.04 | 1.05 | 1.04 | 1.05 | 1.10 | 1.05 |
| Pre-Simulation Emotion | 1.02 | 1.08 | 1.13 | 1.09 | 1.12 | 1.18 | 1.04 |
| PGY | 2.09 | 2.11 | 2.10 | 2.12 | 2.08 | 2.15 | 2.10 |
| Visible Racial Minority | 1.06 | 1.07 | 1.09 | 1.12 | 1.06 | 1.07 | 1.06 |
| Gender | 1.35 | 1.33 | 1.34 | 1.33 | 1.34 | 1.34 | 1.33 |
| **Specialty** |  |  |  |  |  |  |  |
| Internal Medicine | 1.48 | 1.49 | 1.53 | 1.48 | 1.50 | 1.48 | 1.48 |
| Emergency Medicine | 1.56 | 1.59 | 1.59 | 1.56 | 1.60 | 1.59 | 1.58 |
| Critical Care | 2.66 | 2.68 | 2.69 | 2.66 | 2.72 | 2.65 | 2.71 |
| OBGYN | 1.69 | 1.69 | 1.69 | 1.69 | 1.76 | 1.69 | 1.70 |

*Note.* Simulation role refers to participants role in the simulation as team member (assigned a value of 0) or team leader (assigned a value of 1). Pre-simulation emotion refers to the intensity of the emotion of interest measured before the simulation. PGY refers to the post-graduate year of the participant (i.e., training level). Visible racial minority refers to participants’ self-reported identification as either a racialized minority (assigned a value of 0) or a Caucasian (assigned a value of 1). Gender refers to the self-reported gender of the participant (0 = female, 1 = male). All specialty coefficients compare the specialty of interest (assigned a value of 1) to participants specializing in anesthesia (assigned a value of 0). Anesthesia was selected as it had the highest number of observations from all other specializations. OBGYN refers to the obstetrics and gynecology specialty.

**Supplemental Material XIII**

*Variance Inflation Factor Matrix of Variables Used in Multilevel Model Analyses on Perceptions of Performance and Team Mood.*

|  | *Individual Performance* | *Team Performance* | *Team Mood* |
| --- | --- | --- | --- |
| Post-Simulation Emotions |  |  |  |
| Curiosity | 1.48 | 1.48 | 1.48 |
| Confused | 1.83 | 1.83 | 1.83 |
| Ashamed | 2.40 | 2.40 | 2.40 |
| Frustrated | 2.22 | 2.22 | 2.22 |
| Proud | 2.71 | 2.71 | 2.71 |
| Nervous | 2.14 | 2.14 | 2.14 |
| Happy+Hopeful | 3.07 | 3.07 | 3.07 |
| Pre-Simulation Emotions |  |  |  |
| Curiosity | 1.75 | 1.75 | 1.75 |
| Confused | 1.79 | 1.79 | 1.79 |
| Ashamed | 2.34 | 2.34 | 2.34 |
| Frustrated | 1.83 | 1.83 | 1.83 |
| Proud | 2.56 | 2.56 | 2.56 |
| Nervous | 1.96 | 1.96 | 1.96 |
| Happy+Hopeful | 2.67 | 2.67 | 2.67 |
| Simulation Role | 1.19 | 1.19 | 1.19 |
| PGY | 2.26 | 2.26 | 2.26 |
| Specialty |  |  |  |
| Internal Medicine | 1.67 | 1.67 | 1.67 |
| Emergency Medicine | 1.87 | 1.87 | 1.87 |
| Critical Care | 2.61 | 2.61 | 2.61 |
| OBGYN | 1.78 | 1.78 | 1.78 |

*Note.* Simulation role refers to participants role in the simulation as team member (assigned a value of 0) or team leader (assigned a value of 1). PGY refers to the post-graduate year of the participant (i.e., training level). All specialty coefficients compare the specialty of interest (assigned a value of 1) to participants specializing in anesthesia (assigned a value of 0). Anesthesia was selected as it had the highest number of observations from all other specializations. OBGYN refers to the obstetrics and gynecology specialty.

**Supplemental Material XIV**

*Multilevel Models of the Effect of Simulation Role on Post-Simulation Emotions with Covariates, Including Gender and Visible Racial Minority Status.*

|  | ***Curiosity*** | ***Confused*** | ***Ashamed*** | ***Frustrated*** | ***Proud*** | ***Nervous*** | ***Happy and Hopeful*** |
| --- | --- | --- | --- | --- | --- | --- | --- |
| Intercept | 1.212 | 2.634 | 1.331 | 1.463 | 1.272 | 1.714 | -0.318 |
| Simulation role | -0.238 | 0.001 | 0.379 | 0.296 | 0.173 | -0.013 | 0.176 |
| Pre-Simulation Emotion | 0.428 | 0.185 | 0.493 | 0.551 | 0.548 | 0.307 | 0.604 |
| PGY | 0.082 | -0.162 | -0.069 | -0.033 | 0.010 | -0.135 | -0.081 |
| Visible Racial Minority | 0.118 | 0.225 | 0.090 | -0.152 | 0.031 | 0.172 | 0.073 |
| Gender | 0.240 | -0.403 | -0.052 | -0.216 | -0.064 | -0.193 | 0.244 |
| **Specialty** |  |  |  |  |  |  |  |
| Internal Medicine | 0.099 | -0.213 | -0.398 | 0.029 | -0.094 | 0.173 | 0.164 |
| Emergency Medicine | 0.085 | -0.428 | -0.215 | -0.380 | 0.003 | -0.368 | 0.573 |
| Critical Care | -0.582 | 0.055 | -0.477 | -0.208 | -0.091 | 0.530 | 0.622 |
| OBGYN | -0.109 | 0.060 | -0.131 | -0.150 | -0.100 | 0.261 | 0.332 |
| **Variance components** |  |  |  |  |  |  |  |
| Simulation variance | - | 0.291 | 0.218 | - | - | 0.079 | - |
| Team variance | - | - | - | 0.053 | - | - | - |
| Individual variance | 0.084 | 0.033 | 0.026 | 0.102 | <0.001 | 0.162 | 0.023 |

*Note.* Simulation role refers to participants role in the simulation as team member (assigned a value of 0) or team leader (assigned a value of 1). Pre-simulation emotion refers to the intensity of the emotion of interest measured before the simulation. PGY refers to the post-graduate year of the participant (i.e., training level). Visible racial minority refers to participants’ self-reported identification as either a racialized minority (assigned a value of 0) or a Caucasian (assigned a value of 1). Gender refers to the self-reported gender of the participant (0 = female, 1 = male). All specialty coefficients compare the specialty of interest (assigned a value of 1) to participants specializing in anesthesia (assigned a value of 0). Anesthesia was selected as it had the highest number of observations from all other specializations. OBGYN refers to the obstetrics and gynecology specialty. The variance components represent the random effects of the model. Simulation variance refers to the variance estimate at the simulation level. Team variance refers to the variance estimate at the team level. Individual variance refers to the variance estimate at the individual participant level.

**Supplemental Material XV**

*Stata Codes Used for Analyses.*

Comparing team leaders and team members emotions:

- **Curiosity:**
  mixed post_curious leader pre_curious pgy b4.specialty || person_id:
- **Confusion:**
  mixed post_confused leader pre_confused pgy b4.specialty || _all:R.sim_id || person_id:
- **Shame:**
  mixed post_ashamed leader pre_ashamed pgy b4.specialty || _all:R.sim_id || person_id:
- **Frustration:**
  mixed post_frustrated leader pre_frustrated pgy b4.specialty || _all:R.team_id || person_id:
- **Pride:**
  mixed post_proud leader pre_proud pgy b4.specialty || person_id:
- **Nervousness:**
  mixed post_nervous leader pre_nervous pgy b4.specialty || _all:R.sim_id || person_id:
- **Happiness and hopefulness:**
  mixed post_hophap leader pre_hophap pgy b4.specialty || person_id:

Emotions predicting performance and team mood:

- **Individual performance:**
  mixed indperf post_curious post_confused post_ashamed post_frustrated post_hophap post_proud post_nervous pre_curious pre_confused pre_ashamed pre_frustrated pre_hophap pre_proud pre_nervous pgy minor_ind gen leader b4.specialty || _all:R.team_id || person_id:
- **Team performance:**
  mixed tperf post_curious post_confused post_ashamed post_frustrated post_hophap post_proud post_nervous pre_curious pre_confused pre_ashamed pre_frustrated pre_hophap pre_proud pre_nervous pgy minor_ind gen leader b4.specialty || _all:R.sim_id || _all:R.team_id || person_id:

**Team mood:**
mixed tmood post_curious post_confused post_ashamed post_frustrated post_hophap post_proud post_nervous pre_curious pre_confused pre_ashamed pre_frustrated pre_hophap pre_proud pre_nervous pgy minor_ind gen leader b4.specialty || _all:R.sim_id || _all:R.team_id || person_id:

**Suppmental Material XVI**

*Akaike Information Criterion (AIC) and Bayesian Information Criterion (BIC) of Multilevel Models of the Effect of Simulation Role on Post-Simulation Emotions.*

|  | ***Curiosity*** | ***Confused*** | ***Ashamed*** | ***Frustrated*** | ***Proud*** | ***Nervous*** | ***Happy and Hopeful*** |
| --- | --- | --- | --- | --- | --- | --- | --- |
| **Null Model** |  |  |  |  |  |  |  |
| AIC | 520.44 | 492.37 | 464.39 | 502.37 | 475.18 | 432.98 | 578.90 |
| BIC | 529.90 | 504.99 | 477.01 | 514.98 | 484.64 | 445.59 | 588.36 |
| **Predictor-Only Model** |  |  |  |  |  |  |  |
| AIC | 521.34 | 493.79 | 450.29 | 498.06 | 477.14 | 432.81 | 580.84 |
| BIC | 533.95 | 509.56 | 466.05 | 513.83 | 489.76 | 448.58 | 593.46 |
| **Full Model** |  |  |  |  |  |  |  |
| AIC | 505.96 | 492.38 | 424.46 | 478.76 | 426.09 | 405.99 | 516.12 |
| BIC | 537.49 | 527.06 | 459.14 | 513.44 | 454.47 | 440.68 | 547.65 |

**Suppmental Material XVII**

*Akaike Information Criterion (AIC) and Bayesian Information Criterion (BIC) of Multilevel Models of the Effect Of Post-Simulation Emotions on Perceptions of Performance and Team Mood.*

|  | ***Perceptions of Individual Performance*** | ***Perceptions of Team Performance*** | ***Perceptions of Team Mood*** |
| --- | --- | --- | --- |
| **Null Model** |  |  |  |
| AIC | 413.52 | 414.29 | 396.53 |
| BIC | 426.13 | 430.06 | 412.29 |
| **Predictor-Only Model** |  |  |  |
| AIC | 363.13 | 388.22 | 338.47 |
| BIC | 397.82 | 426.06 | 376.31 |
| **Full Model** |  |  |  |
| AIC | 374.92 | 392.95 | 351.30 |
| BIC | 456.90 | 478.09 | 436.44 |

**Supplemental Material XVIII**

*Multilevel Models of the Effect of Post-Simulation Emotions on Perceptions of Individual Performance.*

|  | *Null* | *Null + Post-simulation Emotions* | *Full model with Covariates* |
| --- | --- | --- | --- |
| Intercept | 3.163** | 3.150** | 3.777** |
| Post-Simulation Emotions |  |  |  |
| Curiosity |  | 0.011 | -0.003 |
| Confused |  | -0.073 | -0.108 |
| Ashamed |  | 0.006 | -0.058 |
| Relieved |  | 0.008 | -0.090 |
| Stressed |  | -0.161 | -0.106 |
| Frustrated |  | -0.094 | -0.123 |
| Hopeless |  | -0.066 | -0.008 |
| Happy |  | 0.118 | 0.291* |
| Hopeful |  | -0.074 | -0.066 |
| Proud |  | 0.243** | 0.172 |
| Nervous |  | 0.004 | 0.041 |
| Pre-Simulation Emotions |  |  |  |
| Curiosity |  |  | -0.003 |
| Confused |  |  | 0.068 |
| Ashamed |  |  | 0.013 |
| Relieved |  |  | 0.131 |
| Stressed |  |  | -0.049 |
| Frustrated |  |  | -0.157 |
| Hopeless |  |  | 0.068 |
| Happy |  |  | -0.226 |
| Hopeful |  |  | -0.143 |
| Proud |  |  | 0.186 |
| Nervous |  |  | 0.010 |
| Simulation Role |  |  | 0.045 |
| PGY |  |  | -0.039 |
| Visible Racial Minority |  |  | 0.004 |
| Gender |  |  | -0.212 |
| Specialty |  |  |  |
| Internal Medicine |  |  | -0.156 |
| Emergency Medicine |  |  | -0.022 |
| Critical Care |  |  | -0.182 |
| OBGYN |  |  | -0.020 |
| Variance components |  |  |  |
| Team variance | 0.073 | 0.029 | <0.001 |
| Individual variance | 0.046 | 0.017 | <0.001 |
| ICC_T_ | 0.114 |  |  |
| ICC_I>T_ | 0.186 |  |  |

* *p* < 0.0045, ** *p* < 0.001

*Note.* Simulation role refers to participants role in the simulation as team member (assigned a value of 0) or team leader (assigned a value of 1). PGY refers to the post-graduate year of the participant (i.e., training level). Visible racial minority refers to participants’ self-reported identification as either a racialized minority (assigned a value of 0) or a Caucasian (assigned a value of 1). Gender refers to the self-reported gender of the participant (0 = female, 1 = male). All specialty coefficients compare the specialty of interest (assigned a value of 1) to participants specializing in anesthesia (assigned a value of 0). Anesthesia was selected as it had the highest number of observations from all other specializations. OBGYN refers to the obstetrics and gynecology specialty. The variance components represent the random effects of the model. Team variance refers to the variance estimate at the team level. ICC_T_ refers to the intraclass coefficient for the team level. Individual variance refers to the variance estimate at the individual participant level. ICC_I>T_ refers to the intraclass coefficient for the individual level nested within the team level.

**Supplemental Material XIX**

*Multilevel Models of the Effect of All Post-Simulation Emotions and Stress on Perceptions of Team Mood.*

|  | *Null* | *Null + Post-simulation Emotions* | *Full model with Covariates* |
| --- | --- | --- | --- |
| Intercept | 3.163** | 3.730** | 3.933** |
| Post-Simulation Emotions |  |  |  |
| Curiosity |  | -0.009 | -0.006 |
| Confused |  | -0.096 | -0.065 |
| Ashamed |  | -0.045 | -0.145 |
| Relieved |  | 0.015 | 0.021 |
| Stressed |  | -0.123 | -0.102 |
| Frustrated |  | -0.122 | -0.167 |
| Hopeless |  | 0.015 | 0.090 |
| Happy |  | 0.195* | 0.247* |
| Hopeful |  | 0.015 | 0.046 |
| Proud |  | 0.066 | 0.054 |
| Nervous |  | 0.045 | -0.024 |
| Pre-Simulation Emotions |  |  |  |
| Curiosity |  |  | -0.045 |
| Confused |  |  | 0.041 |
| Ashamed |  |  | 0.194 |
| Relieved |  |  | -0.021 |
| Stressed |  |  | 0.060 |
| Frustrated |  |  | 0.076 |
| Hopeless |  |  | -0.258 |
| Happy |  |  | -0.011 |
| Hopeful |  |  | 0.058 |
| Proud |  |  | -0.132 |
| Nervous |  |  | -0.028 |
| Simulation Role |  |  | 0.031 |
| PGY |  |  | -0.070 |
| Visible Racial Minority |  |  | -0.053 |
| Gender |  |  | -0.027 |
| Specialty |  |  |  |
| Internal Medicine |  |  | -0.069 |
| Emergency Medicine |  |  | 0.202 |
| Critical Care |  |  | 0.162 |
| OBGYN |  |  | 0.278 |
| Variance components |  |  |  |
| Simulation variance | 0.085 | 0.063 | 0.047 |
| Team variance | 0.025 | <0.001 | <0.001 |
| Individual variance | 0.044 | 0.018 | 0.008 |
| ICC_S_ | 0.150 |  |  |
| ICC_T>S_ | 0.194 |  |  |
| ICC_I>T>S_ | 0.271 |  |  |

* p < 0.0045, ** p < 0.001

*Note.* Simulation role refers to participants role in the simulation as team member (assigned a value of 0) or team leader (assigned a value of 1). PGY refers to the post-graduate year of the participant (i.e., training level). Visible racial minority refers to participants’ self-reported identification as either a racialized minority (assigned a value of 0) or a Caucasian (assigned a value of 1). Gender refers to the self-reported gender of the participant (0 = female, 1 = male). All specialty coefficients compare the specialty of interest (assigned a value of 1) to participants specializing in anesthesia (assigned a value of 0). Anesthesia was selected as it had the highest number of observations from all other specializations. OBGYN refers to the obstetrics and gynecology specialty. The variance components represent the random effects of the model. Simulation variance refers to the variance estimate at the simulation level. ICC_S_ refers to the intraclass coefficient for the simulation level. Team variance refers to the variance estimate at the team level. ICC_T>S_ refers to the intraclass coefficient for the team level nested within the simulation level. Individual variance refers to the variance estimate at the individual participant level. ICC_I>T>S_ refers to the intraclass coefficient for the individual level nested within the team level and simulation level.

**Supplemental Material XX**

*Multilevel Models of the Effect of Post-Simulation Emotions on Perceptions of Team Performance.*

|  | *Null* | *Null + Post-simulation Emotions* | *Full model with Covariates* |
| --- | --- | --- | --- |
| Intercept | 3.577** | 3.417** | 4.347** |
| Post-Simulation Emotions |  |  |  |
| Curious |  | -0.043 | -0.032 |
| Confused |  | -0.067 | -0.122 |
| Ashamed |  | -0.029 | -0.071 |
| Relieved |  | 0.089 | -0.001 |
| Stressed |  | -0.124 | -0.113 |
| Frustrated |  | -0.052 | 0.008 |
| Hopeless |  | -0.171 | -0.182 |
| Happy |  | 0.261* | 0.372 |
| Hopeful |  | -0.065 | -0.017 |
| Proud |  | 0.009 | -0.025 |
| Nervous |  | 0.133 | 0.069 |
| Pre-Simulation Emotions |  |  |  |
| Curious |  |  | -0.037 |
| Confused |  |  | 0.148 |
| Ashamed |  |  | 0.006 |
| Relieved |  |  | -0.036 |
| Stressed |  |  | -0.118 |
| Frustrated |  |  | -0.218 |
| Hopeless |  |  | 0.035 |
| Happy |  |  | -0.225 |
| Hopeful |  |  | 0.072 |
| Proud |  |  | 0.089 |
| Nervous |  |  | 0.101 |
| Simulation Role |  |  | 0.093 |
| PGY |  |  | -0.150 |
| Visible Racial Minority |  |  | 0.006 |
| Gender |  |  | -0.203 |
| Specialty |  |  |  |
| Internal Medicine |  |  | -0.162 |
| Emergency Medicine |  |  | 0.158 |
| Critical Care |  |  | 0.244 |
| OBGYN |  |  | 0.379 |
| Variance components |  |  |  |
| Simulation variance | 0.064 | 0.073 | 0.051 |
| Team variance | 0.110 | 0.036 | 0.012 |
| Individual variance | 0.147 | 0.087 | 0.069 |
| ICC_S_ | 0.091 |  |  |
| ICC_T>S_ | 0.249 |  |  |
| ICC_I>T>S_ | 0.384 |  |  |

* *p* < 0.0045, ** *p* < 0.001

*Note.* Simulation role refers to participants role in the simulation as team member (assigned a value of 0) or team leader (assigned a value of 1). PGY refers to the post-graduate year of the participant (i.e., training level). Visible racial minority refers to participants’ self-reported identification as either a racialized minority (assigned a value of 0) or a Caucasian (assigned a value of 1). Gender refers to the self-reported gender of the participant (0 = female, 1 = male). All specialty coefficients compare the specialty of interest (assigned a value of 1) to participants specializing in anesthesia (assigned a value of 0). Anesthesia was selected as it had the highest number of observations from all other specializations. OBGYN refers to the obstetrics and gynecology specialty. The variance components represent the random effects of the model. Simulation variance refers to the variance estimate at the simulation level. ICC_S_ refers to the intraclass coefficient for the simulation level. Team variance refers to the variance estimate at the team level. ICC_T>S_ refers to the intraclass coefficient for the team level nested within the simulation level. Individual variance refers to the variance estimate at the individual participant level. ICC_I>T>S_ refers to the intraclass coefficient for the individual level nested within the team level and simulation level.

**References**

Ahn, B. (Tony), Maurice-Ventouris, M., Bilgic, E., Yang, A., Lau, C. H.-H., Peters, H., Li, K., Chang-Ou, D., & Harley, J. M. (2023). A scoping review of emotions and related constructs in simulation-based education research articles. *Advances in Simulation*, *8*(1), 22. https://doi.org/10.1186/s41077-023-00258-z

American Educational Research Association, American Psychological Association, & National Council on Measurement in Education (Eds.). (2014). *Standards for Educational and Psychological Testing*. American Educational Research Association.

Bakhtiar, A., Webster, E. A., & Hadwin, A. F. (2018). Regulation and socio-emotional interactions in a positive and a negative group climate. *Metacognition and Learning*, *13*(1), 57–90. https://doi.org/10.1007/s11409-017-9178-x

Colbert, C. Y., Brateanu, A., Nowacki, A. S., Prelosky-Leeson, A., & French, J. C. (2021). An Examination of Resident Perspectives on Survey Participation and Methodology: Implications for Educational Practice and Research. *Journal of Graduate Medical Education*, *13*(3), 390–403. https://doi.org/10.4300/JGME-D-20-01431.1

Duffy, M. C., Lajoie, S. P., Pekrun, R., & Lachapelle, K. (2020). Emotions in medical education: Examining the validity of the Medical Emotion Scale (MES) across authentic medical learning environments. *Learning and Instruction*, *70*, 101150. https://doi.org/10.1016/j.learninstruc.2018.07.001

Fontaine, J. J. R., Scherer, K. R., & Soriano, C. (Eds.). (2013). *Components of Emotional Meaning: A sourcebook* (1st ed.). Oxford University Press. https://doi.org/10.1093/acprof:oso/9780199592746.001.0001

Frumos, F.-V., Leonte, R., Candel, O. S., Ciochină-Carasevici, L., Ghiaţău, R., & Onu, C. (2024). The relationship between university students’ goal orientation and academic achievement. The mediating role of motivational components and the moderating role of achievement emotions. *Frontiers in Psychology*, *14*. https://doi.org/10.3389/fpsyg.2023.1296346

Gogol, K., Brunner, M., Goetz, T., Martin, R., Ugen, S., Keller, U., Fischbach, A., & Preckel, F. (2014). “My Questionnaire is Too Long!” The assessments of motivational-affective constructs with three-item and single-item measures. *Contemporary Educational Psychology*, *39*(3), 188–205. https://doi.org/10.1016/j.cedpsych.2014.04.002

Graham, A. J., McCormack, T., Lorimer, S., Hoerl, C., Beck, S. R., Johnston, M., & Feeney, A. (2023). Relief in everyday life. *Emotion*, *23*(7), 1844–1868. https://doi.org/10.1037/emo0001191

Gross, J. J. (2024). Conceptual foundations of emotion regulation. In J.J. Gross & B. Ford (Eds.), *Handbook of emotion regulation, 3rd ed* (pp. 3–12). The Guilford Press (Taylor & Francis Group).

Harley, J. M., Bouchet, F., Hussain, M. S., Azevedo, R., & Calvo, R. A. (2015). A Multi-Componential Analysis of Emotions during Complex Learning with an Intelligent Multi-agent System. *Computers in Human Behavior*, *48*, 615–625. https://doi.org/10.1016/j.chb.2015.02.013

Harley, J. M., & Pekrun, R. (2024). Managing Emotions in Education: The Emotion Regulation in Achievement Situations Model. In J.J. Gross & B. Ford (Eds.), *Handbook of Emotion Regulation* (3rd ed., pp. 536–544). The Guilford Press (Taylor & Francis Group).

Harley, J. M., Pekrun, R., Taxer, J. L., & Gross, J. J. (2019). Emotion Regulation in Achievement Situations: An Integrated Model. *Educational Psychologist*, *54*(2), 106–126. https://doi.org/10.1080/00461520.2019.1587297

Jarrell, A., & Lajoie, S. P. (2017). The regulation of achievements emotions: Implications for research and practice. *Canadian Psychology / Psychologie Canadienne*, *58*(3), 276–287. https://doi.org/10.1037/cap0000119

Joseph, M., Ray, J. M., Chang, J., Cramer, L. D., Bonz, J. W., Yang, T. J., Wong, A. H., Auerbach, M. A., & Evans, L. V. (2022). All clinical stressors are not created equal: Differential task stress in a simulated clinical environment. *AEM Education and Training*, *6*(2), e10726. https://doi.org/10.1002/aet2.10726

Kim, J., Neilipovitz, D., Cardinal, P., Chiu, M., & Clinch, J. (2006). A pilot study using high-fidelity simulation to formally evaluate performance in the resuscitation of critically ill patients: The University of Ottawa Critical Care Medicine, High-Fidelity Simulation, and Crisis Resource Management I Study. *Critical Care Medicine*, *34*(8), 2167. https://doi.org/10.1097/01.CCM.0000229877.45125.CC

LeBlanc, V. R., & Posner, G. D. (2022). Emotions in simulation-based education: Friends or foes of learning? *Advances in Simulation*, *7*(1), 3. https://doi.org/10.1186/s41077-021-00198-6

Li, C. (2013). Little’s Test of Missing Completely at Random. *The Stata Journal*, *13*(4), 795–809. https://doi.org/10.1177/1536867X1301300407

Little, R. J. A. (1988). A Test of Missing Completely at Random for Multivariate Data with Missing Values. *Journal of the American Statistical Association*, *83*(404), 1198–1202. https://doi.org/10.1080/01621459.1988.10478722

Lyubomirsky, S., & Kurtz, J. (2009). Happiness. In D. Sander & K. Scherer (Eds.), *The Oxford companion to emotion and the affective sciences.* Oxford University Press. https://awspntest.apa.org/record/2009-16563-000

Pekrun, R. (2006). The Control-Value Theory of Achievement Emotions: Assumptions, Corollaries, and Implications for Educational Research and Practice. *Educational Psychology Review*, *18*(4), 315–341. https://doi.org/10.1007/s10648-006-9029-9

Pekrun, R. (2024). Control-Value Theory: From Achievement Emotion to a General Theory of Human Emotions. *Educational Psychology Review*, *36*(3), 1–36. https://doi.org/10.1007/s10648-024-09909-7

Pekrun, R., Goetz, T., Frenzel, A. C., Barchfeld, P., & Perry, R. P. (2011). Measuring emotions in students’ learning and performance: The Achievement Emotions Questionnaire (AEQ). *Contemporary Educational Psychology*, *36*(1), 36–48. https://doi.org/10.1016/j.cedpsych.2010.10.002

Pekrun, R., Marsh, H. W., Elliot, A. J., Stockinger, K., Perry, R. P., Vogl, E., Goetz, T., Van Tilburg, W. A. P., Lüdtke, O., & Vispoel, W. P. (2023). A three-dimensional taxonomy of achievement emotions. *Journal of Personality and Social Psychology*, *124*(1), 145–178. https://doi.org/10.1037/pspp0000448

Pekrun, R., & Stephens, E. J. (2010). Achievement Emotions in Higher Education. In *Higher Education: Handbook of Theory and Research* (pp. 257–306). Springer, Dordrecht. https://doi.org/10.1007/978-90-481-8598-6_7

Pekrun, R., Vogl, E., Muis, K. R., & Sinatra, G. M. (2017). Measuring emotions during epistemic activities: The Epistemically-Related Emotion Scales. *Cognition and Emotion*, *31*(6), 1268–1276. https://doi.org/10.1080/02699931.2016.1204989

Shuman, V., & Scherer, K. R. (2013). Concepts and Structures of Emotions. In *International Handbook of Emotions in Education*. Routledge. https://doi.org/10.4324/9780203148211.ch2

Taber, K. S. (2018). The Use of Cronbach’s Alpha When Developing and Reporting Research Instruments in Science Education. *Research in Science Education*, *48*(6), 1273–1296. https://doi.org/10.1007/s11165-016-9602-2

Thibault, L.-P., Bourque, C. J., Luu, T. M., Huot, C., Cardinal, G., Carriere, B., Dupont-Thibodeau, A., & Moussa, A. (2022). Residents as Research Subjects: Balancing Resident Education and Contribution to Advancing Educational Innovations. *Journal of Graduate Medical Education*, *14*(2), 191–200. https://doi.org/10.4300/JGME-D-21-00530.1

Vogl, E., Pekrun, R., Murayama, K., & Loderer, K. (2020). Surprised–curious–confused: Epistemic emotions and knowledge exploration. *Emotion*, *20*(4), 625–641. https://doi.org/10.1037/emo0000578

Watson, D., Clark, L. A., & Tellegen, A. (1988). Development and validation of brief measures of positive and negative affect: The PANAS scales. *Journal of Personality and Social Psychology*, *54*(6), 1063. https://doi.org/10.1037/0022-3514.54.6.1063
